# Taking a look accurately at the alteration of interfacial asphaltene film exposed to the ionic surfactants as demulsifiers

**DOI:** 10.1038/s41598-023-39731-0

**Published:** 2023-08-08

**Authors:** Soheila Javadian, S. Morteza Sadrpoor, Mahnaz Khosravian

**Affiliations:** Department of Physical Chemistry, Faculty of Basic Science, Tarbiat Modares University, P.O. Box 14115-175, Tehran, Islamic Republic of Iran

**Keywords:** Chemistry, Surface chemistry

## Abstract

The water droplets surrounded by a rigid interfacial asphaltene (ASP) film is one of the major setbacks in the petroleum industry. In this study, the properties of the interfacial ASP films around water droplets exposed to ionic surfactants as demulsifier were investigated. According to molecular dynamics (MD) simulation, the anionic surfactants are more effective than the cationic surfactant in the demulsification process since the anionic surfactants have the exact desire to localize not only near the ASP molecules but also near the water molecules. It has been found that it is likely to cause film changes and ruptures. Also, the MD simulation results for the desired surfactant, anionic surfactant, demonstrated that an increase in the surfactant concentration had an adverse effect on the system by hindering the change in the interfacial film. The increase in the temperature along with the enhancement in the adsorption rate of the surfactant results in the better performance of the demulsifier. Taking the MD and quantum results into account, the film deformation is a decisive factor in demulsification. The quantum computation has indicated that the electrostatic interactions play a significant role in selecting the attraction position and adsorption energy of the surfactant molecules.

## Introduction

Even though the energy resource revolution has become more common these days, the use of crude oil, as a well-known energy resource, ranks as one of the most significant. The formation of water-in-crude oil (W/O) emulsion resulting from well-production is one of the major obstacles in the petroleum industry^1,2^. Crude oil involving water as an impurity surrounded by indigenous oil surfactants, especially asphaltene (ASP) molecule, takes its toll on transportation, corrosion^3^, storage, and catalyst poisoning^4^. Therefore, some demulsification approaches in the oil refining process, which include electrical, mechanical, and chemical methods, are carried out to coalesce the individual water droplets and separate them from the crude oil phase^5^. Among the above-mentioned methods, chemical methods have attracted the attention of researchers since they are more economical and they cushion the devastating effect on the crude oil phase^5^. Since the surfactants can replace the ASP in the rigid film as a coalescence inhibitor of the water droplets, and cause a change in film properties^6^, countless research studies have been carried out into the interfacial film essence and the effects of the structural properties of both ASP and oil^7,8^. After recognizing the physical properties of interfacial films^6,9^, some scientists were urged to eradicate the films with more sufficient demulsifiers^4^. It means that demulsifiers reduce the stability of the emulsion and the water extracted from crude oil increases. Some experimental investigations proved that surfactants, in both ionic^10^ and neutral forms^11^, and polymers^12^ play a significant role in the demulsification process of crude oil.

From the molecular dynamics simulation, MD, point of view, the arrangements of the individual content at the interfacial film were explored to confirm the construction of the interfacial film in the presence of disparate demulsifiers^13,14^. It has been concluded that ionic demulsifiers and polymers have a tendency to be adsorbed on the interfacial film and change the ASP arrangements compared to their initial arrangement^15^. In addition, more proper arrangements concerning the available contents in the interface were examined from the quantum mechanics standpoint^16^.

Despite the substantial increase in using adequate demulsifiers, there are some unanswered questions. For instance, how different demulsifiers can alter the structure of an interfacial film. Therefore, it is important to know whether predicting a demulsifier is plausible or not. Therefore, in this work, an MD simulation study was carried out to trace the root of the change in the interfacial film caused by the demulsification process under different conditions such as the type of surfactant, concentration, and the effect of temperature, which were less investigated in previous studies. Next, a quantum mechanics study was carried out to demonstrate the interaction energy between the individual contents. Also, the changes in HOMO and LUMO energy of the ASP were studied before and after adding the surfactants. Additionally, the trend in the computational prediction was proven by an experimental study.

## Materials and methods

### MD simulation method

**Figure 1:**
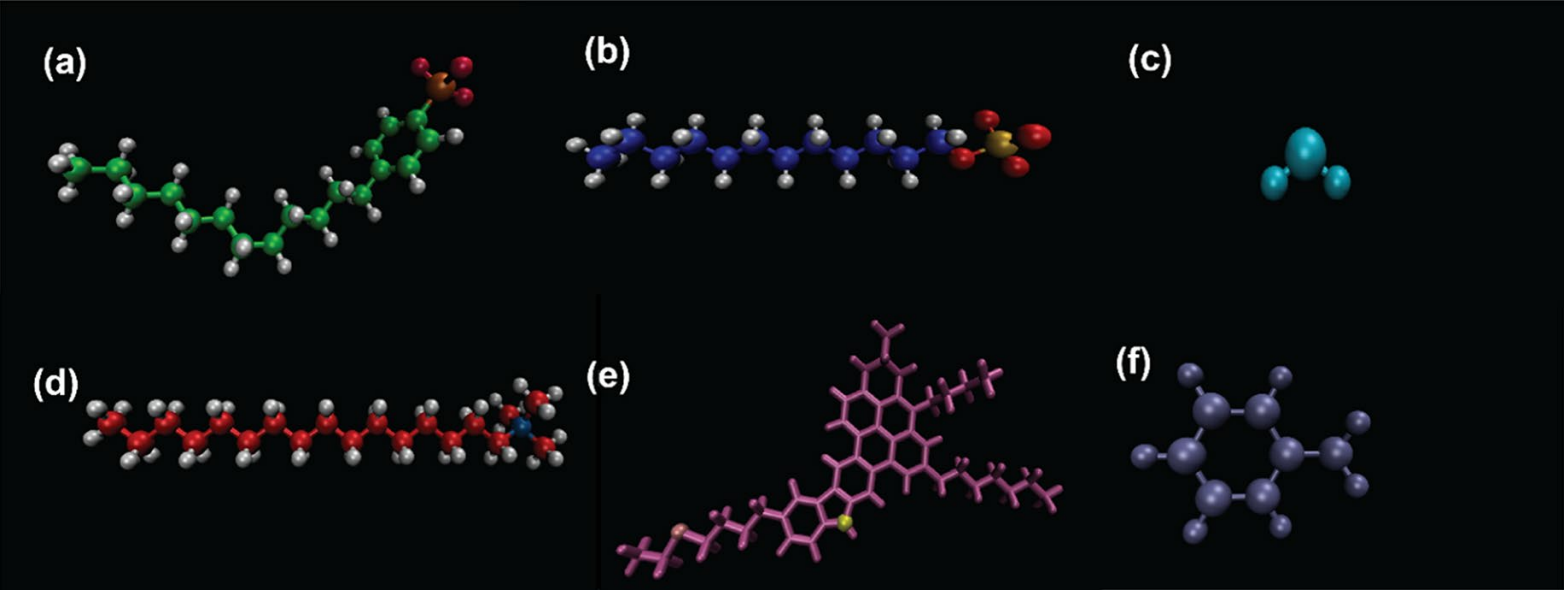
The three-dimensional chemical structures of molecules used in the simulations. SDBS (C_18_H_29_NaO_3_S) (**a**), SDS (C_12_H_25_NaSO_4_) (**b**), H_2_O (**c**), CTAB (C_19_H_42_BrN) (**d**), petroleum ASP (C_47_H_55_NS) yellow and orange circles depict N and S respectively (**e**), Toluene (C_7_H_8_). The counter ion of ionic surfactants was omitted.

All contents of systems such as ASP, water, toluene, and surfactant molecules demonstrated in Fig. 1 were embedded in a simulation box using packmol^17^. For the initial structure, a water droplet was put into the box center, and it was encapsulated by an ASP shell at a certain distance^4^. Then, the box was filled with toluene molecules. The reason for using petroleum ASP is attributed to its properties, including heteroatoms located in different parts of the molecule, peripheral substitution, and the benzene rings that are fused together^18^. In the following, the prepared box was initially simulated in the NPT ensemble at T = 298 K and P = 1 atm with the time step of 2 fs for 6 ns, and then its output was employed as an initial structure in other parts. Later, the surfactant molecules were added to the previous output to set up an initial situation. The TIP3P model was implemented on the water molecules^19^. The simulations were performed by means of NAMD^20^. CHARMM General Force Field (CGenff) was employed for all contents of the system^21^. The periodic boundary condition was employed in three directions, namely x, y, and z. The Lenard-jones potential was applied to make a non-bonded interaction model, and the Vander Waals attraction and steric repulsion were achieved with the cut off of 12 Å^22^. The long-range electrostatic force was calculated using the Particular Mesh Ewald (PME) method^23^. In order to verify the force field accuracy, the diffusion coefficient for a toluene box was calculated and the result was given in Figure S1. All properties of the system were also given in Table S1.

### Quantum calculation

The geometric optimization was performed for ASP and all surfactants using the Gaussian 09 program and density functional theory (DFT) method with M062X density functions and 6-311g(d,p) basis set. The adsorption energies were calculated as follows

1 $${E}_{adsorption}= {E}_{asphaltene-surfactant}-\left({E}_{surfactant}+{E}_{asphaltene}\right)$$

The molecular electrostatic surface potential (ESP) was plotted using GaussView 6. The atom in the molecule (AIM) method, through AIM 2000 package, was employed to evaluate the hydrogen bonding, and the energy of the hydrogen bonding (${E}_{HB}$) was estimated similar to our previous study (Eq. 3)^24^, where $V\left(r\right)$ signifies the value for a local potential energy Moreover, as reported in our previous study^25^, the energy gap $({E}_{g})$, electronic chemical potential $(\mu )$, and chemical hardness $(\eta )$ were calculated using Eqs. (3–5):

2 $$\text{The energy of Hydrogen bonding }\left({E}_{HB}\right)\, {E}_{HB}=0.5 V\left(r\right)$$

3 $$\text{Energy gap }({E}_{g})\, {E}_{g}={E}_{LUMO}-{E}_{HOMO}$$

4 $$\text{Electronic chemical po tential }\left(\mu \right) \mu =\frac{{E}_{LUMO}+{E}_{HOMO}}{2}$$

5 $$\text{Chemical hardness }(\eta )\,\eta =\frac{{E}_{LUMO}-{E}_{HOMO}}{2}$$

### Materials

The chemical materials used in the current study include sodium dodecyl sulfate (Merck, purity 98%), cetrimonium bromide (CTAB), and sodium dodecyl benzene sulfonate (Aldrich, technical grade). Moreover, the dead crude oil (API = 17.4, viscosity = 37.7 cP) was taken from the Iranian reservoir.

### The crude oil characterization

The percentage of various components of the crude oil such as saturate, aromatic, resin, and asphaltene (SARA), was determined using the ASTM D-2007 method. The results of the SARA analysis are represented in Table S2. Also, the viscosity of crude oil was measured at 298 K (Table S2). The results have already been reported in the previous study^26^.

### The preparation of water in crude oil emulsion

The distilled water was gradually added to the crude oil (the proportion of crude oil to water was 3:2 V/V), and the mixture was stirred at 1100 rpm for 110 min. The water was not separated from the W/O emulsion over 24 h.

### Dehydration method

A customary method for evaluating the demulsifier’s performance is the bottle test^26,27^. The formed W/O emulsion was poured into the graded bottles (at room temperature), and then the surfactants (as demulsifiers) were added to the stable emulsion, and the water precipitated from the emulsion was noted.

The demulsification efficiency was calculated by Eq. (6), where ${V}_{0}$ and ${V}_{\text{s}}$ are the initial volume and separated water, respectively.

6 $$E\text{\%}=\frac{{V}_{s}}{{V}_{0}} \times 100$$

To study the effect of temperature on the demulsification performance, the more effective demulsifier was selected. After that, the function of the selected demulsifier was evaluated at different temperatures (298, 323, and 348 K). It is noteworthy that other conditions such as the formation of W/O emulsion, are the same as mentioned above.

## Result and discussion

### MD simulation

#### Effect of demulsifier intrinsic features

**Figure 2:**
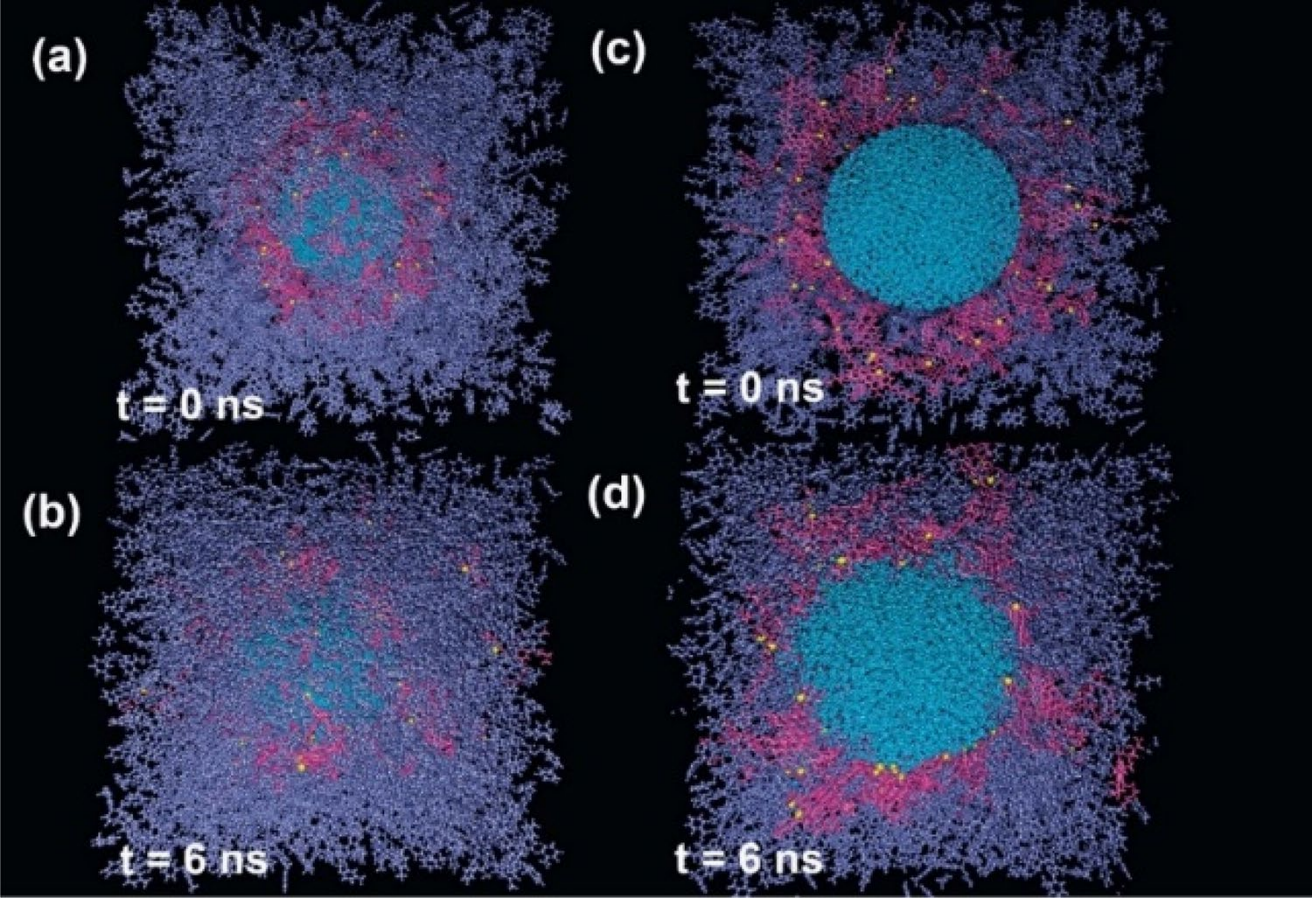
The snapshot of the initial configuration of the system containing all contents other than surfactant molecule (demulsifier) (**a**), and its side view (**c**). The final structure of the system (**b**), and its side view (**c**). Color code: color schemes are the same as Fig. 1.

As a first step, to find out about the dimension of interfacial ASP film, several ASP molecules were put around the water droplet (Fig. 2a,c). After the calculation, the organized interfacial film stemmed from ASPs adsorption indicates self-aggregation among ASPs at some parts^4^ (Fig. 2b,d). In general, asphaltenes tend to self-aggregate, this behavior has also been seen in other systems containing asphaltenes^28^. If the interfacial film settled between two phases is assumed as a 2D film, a fence-like structure appears. However, an utterly uniform film was not observed. The result is consistent with the results approved by the Cadena-Nava RD team^29^. By characterizing 2D and 3D structures in the ASP accumulations, they revealed the dimensions of the interfacial film and highlighted their importance.

In the following, anionic and cationic surfactants as demulsifiers are separately added around the interfacial film obtained from the previous step to determine what happens to the interfacial film after exposure to various demulsifiers. The interfacial film features related to demulsifier-added systems were compared with the film features without any demulsifiers. Density is one of the critical physical quantities to depict the system content distribution^30,31^. Therefore, the density profile in the direction of the Z-axis is utilized to determine the transformation of interfacial ASP film based on the contents distribution variation^20,32^.

**Figure 3:**
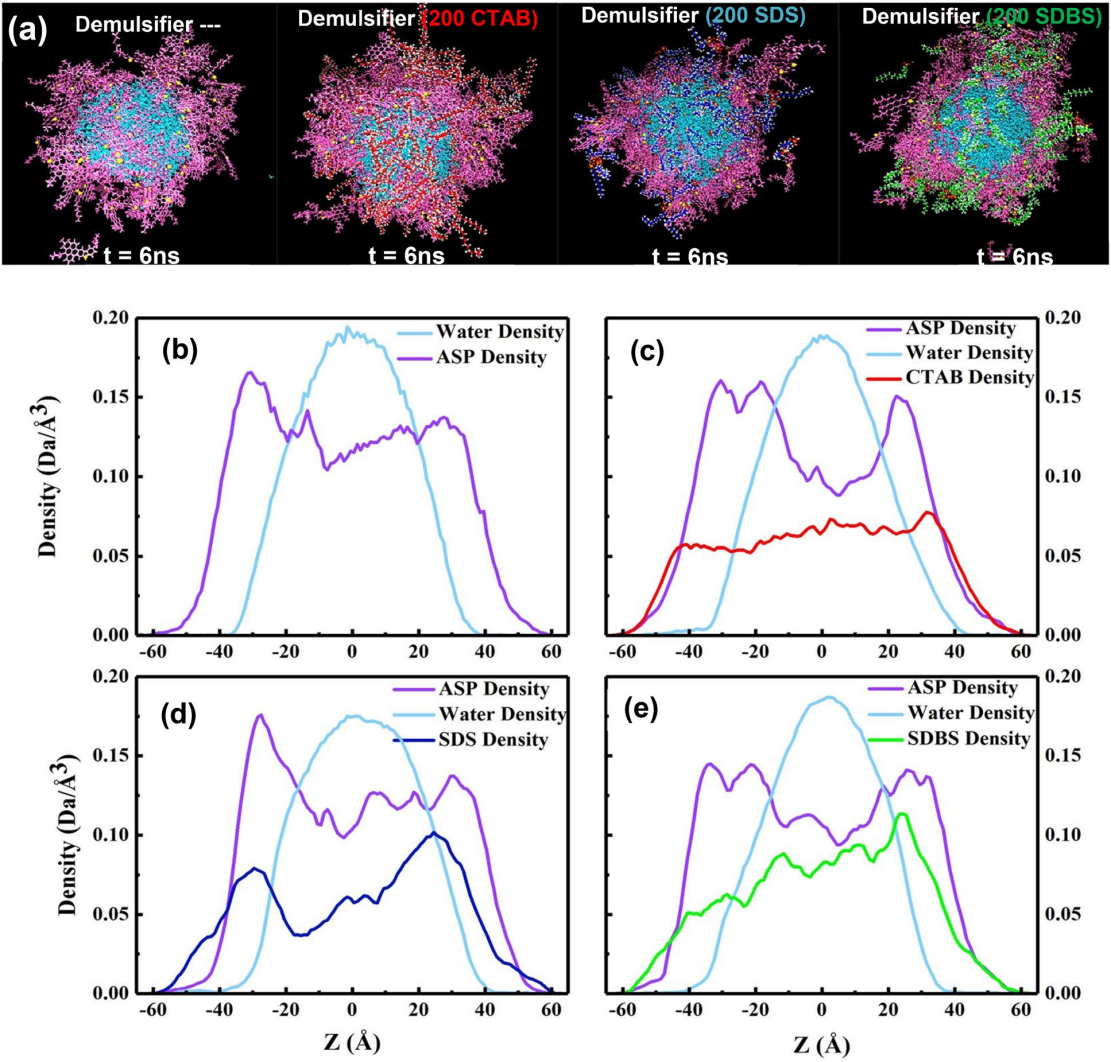
The snapshot of the final structure of four systems with non-surfactant, CTAB, SDS, SDBSand as demulsifier, from left to right (**a**). Color code: color schemes are the same as Fig. 1. The density profile of some contents of the four mentioned systems (**b**–**e**).

The observed plots in the density of water (Fig. 3b–e) are appropriate evidence to approve that the spherical structure of water droplets remained moderately stable. By looking at Figure S2, it can be seen that by adding surfactants to systems, the density figures deviate from the figures of the non-surfactant system. This variation shows that ASP film has gone through a rearrangement^5^, thereby causing uncovered parts onto the surface of the water droplets. Underlying causes of appearing asp-uncovered part of droplets are the reduction of the ASP molecule distance and more aggregation at the position of maximum fluctuation^33^. According to the density profiles (Fig. 3c–e), the cationic surfactant, CTAB, did it better than others. But in the following, it will be revealed why the CTAB molecule is not as effective as anionic demulsifiers.

**Figure 4:**
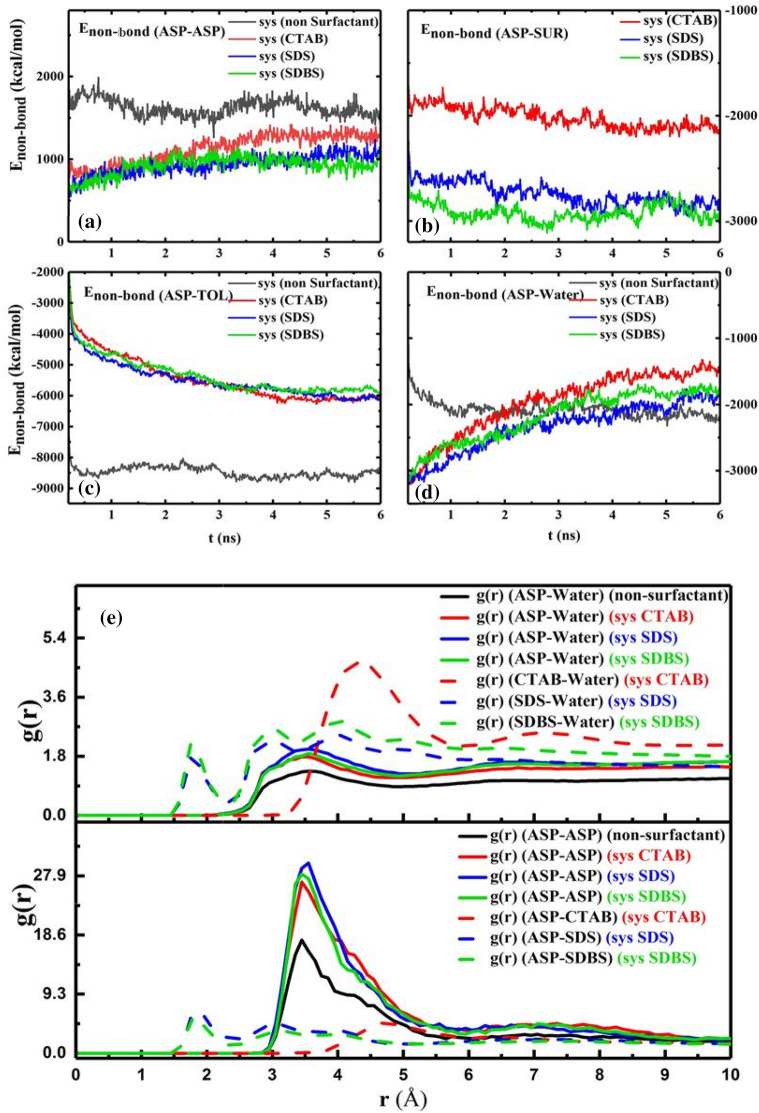
The plot of E_non-bond_ between some pair contents of the four systems with non-surfactant, CTAB, SDS, SDBS as demulsifier (**a**–**d**). The RDF of the systems containing different demulsifier (**e**).

The snapshot of the water droplet (Fig. 3a) shows that the demulsifiers make the water droplet uncover and the interfacial film rupture more^34^, simultaneously. All changes in the ASP film are considered film transformations instead of a film eradication. Other physical quantities such as non-bond energy^35^ (Enon-bond), radial distribution function^18^ (RDF or g(r)), mean square displacement (MSD), and diffusion coefficient (D)^4^ were calculated to estimate the efficiency of demulsifiers. The E_non-bond_ is divided into two parts:, namely van der Waals interaction energy (E_VDW_) and electrostatic interaction energy (E_ele_). The contribution of each term affects the E_non-bond_. The E_non-bond_ between pair contents is used to more accurately predict the film alteration (Fig. 4a–d). In the presence of the demulsifier, the value of absolute E_non-bond_ between ASP–ASP decreases, and also the distance between them declines. The change in distance will be indicated by the g(r) intensity variation in the following. The contribution of the E_non-bond_ is shown in Figure S3. It is noticeable that the more the absolute E_non-bond_ between the ASP–SUR, the less the absolute E_non-bond_ between the ASP-ASP molecules would be. This reverse trend is quite observable in Fig. 4a,b. Figure 4b depicts a higher attraction energy between the anionic surfactants and the ASP molecules, showing the greater tendency of the anionic surfactants to be near the ASP molecules. Also, by adding the surfactants to the systems, the E_non-bond_ of the ASP molecules with both oil and water phase decreases Fig. 4c,d. The redial distribution function (g(r)) shows the distance of the shell surrounding a certain molecule^36^.

Figure 4e shows that by embedding surfactants into the interfacial film, the intensity of the first peak of g(r)_ASP-ASP_, at $r=$ 3.45 Å, there is a decrease in the distance of ASPs^18,37^, and the first peak of g(r)_ASP-SUR_ corresponds to the hydrogen bonding calculated in the quantum section. The amount of g(r) of CTAB-ASP indicates that the distance between CTAB and ASP molecule is greater than the distance between the anionic surfactant and ASP^38^. So, the first reason for the different performance of cationic and anionic surfactants can be traced to the difference in their tendency to select adsorption sites on the interfacial film. All the results can be justified by the tendency of anionic surfactants’ head group to be adsorbed next to the positively charged hydrogen bonded to the ASP heteroatom, i.e., N atom. These results are consistent with both E_non-bond_ between the surfactants and the ASP molecules and the interaction energy obtained in the quantum section.

**Figure 5:**
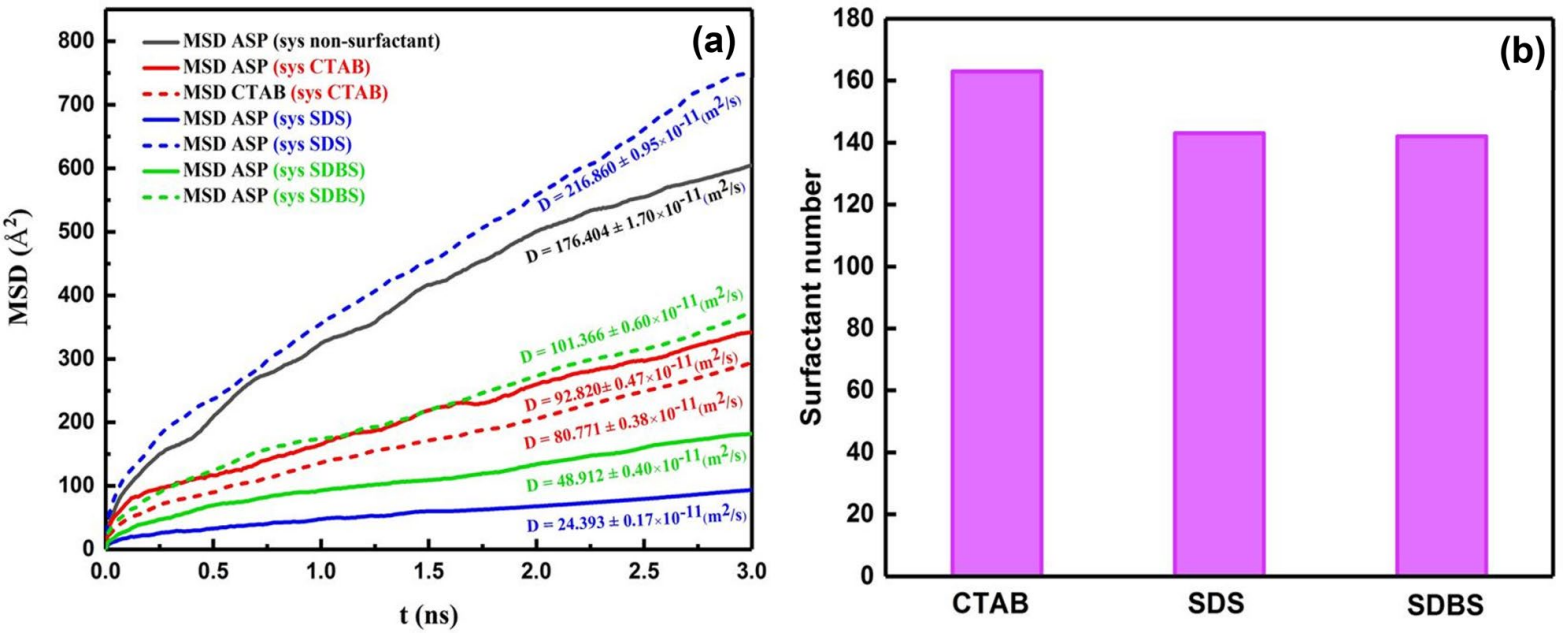
The mean squared displacement and its slope (diffusion coefficient) which belong to ASP and demulsifier molecules for the systems with different demulsifiers (**a**). Distribution histogram of the number of demulsifier molecules adsorbed onto the water droplet (**b**).

It was mentioned above that demulsifiers were added to the output of the first calculation, which was the system without demulsifiers (Fig. 2b,d), so after adding the surfactant, the value of MSD of ASPs shows the displacement of the ASP molecules present in the ASP film (Fig. 5a). Therefore, the more the ASP molecule displacement occurs, the more alteration will be observed in the film structure. Figure 5a depicts that the most value of MSD and its slope^39^ (diffusion coefficient) belong to the system containing CTAB surfactant, which is consistent with the density profile (Figure S1). It means that the most variation related to the density profile of ASP is consistent with the trend of MSD and diffusion coefficient in the system including CTAB as a demulsifier. According to these results, it is expected that the performance of the CTAB surfactant will be higher than the performance of anionic surfactants. But, in addition, the number of adsorbed surfactants on the water surface can significantly predict which type of surfactant can be more efficient in high-yielding demulsification. Although CTAB molecules contribute to more rearrangement of the film, the number of CTAB molecules adsorbed onto the water surface causes a circumstance that either seals or drains away the water droplets.

Figure 5b displays the more CTAB molecules adsorbed on water. It is explained that the more the demulsifiers are adsorbed on the water droplet, the more the rupture should be sealed. Therefore, this phenomenon can constrain draining water away from the droplet to adjacent droplets and also hamper the formation of bigger surrounded droplets compared to the case of anionic demulsifiers. Although g(r) peak positions (Fig. 4e) and E_int_ (Table S3) of anionic surfactants show that they have formed more robust hydrogen bonds with water molecules in comparison to the cationic ones, E_ad_ in the quantum section conveys that anionic surfactant desire to be near the ASP molecule. The length of the hydrogen bond was confirmed by the available peak at $r$ ≈ 2 Å (Fig. 4e) and Table S4. Despite the more robust hydrogen bond between anionic demulsifiers and water, the tendency of the anionic surfactant to be adsorbed near the ASP molecule can prevent the water droplet from being sealed. The results of this section cast a positive light on the way of selecting more effective demulsifiers and can pave the way for anticipating that anionic surfactants, SDS and SDBS, can be more adequate demulsifiers than cationic surfactants (CTAB). Because not only do they interact with the ASP molecules and transform the interfacial film, but also they make ruptures in the interfacial film and preserve them. All of these are conducive to creating a better demulsification function. In addition, among the anionic surfactants, SDS and SDBS, SDBS has been considered to be a more efficient demulsifier because of the benzene ring in its structure. The π- π interaction between ASP and SDBS helps SDBS stay near the ASP molecule. According to the research investigation carried out by Ming Duan^2^ the effective demulsifier tends to settle in the vicinity of not only water but also the ASP molecules. Therefore, SDBS has been selected as the desired surfactant, which was confirmed by the experimental results obtained in the bottle test (Fig. 12).

#### Observed trend of increasing demulsifier concentration

**Figure 6:**
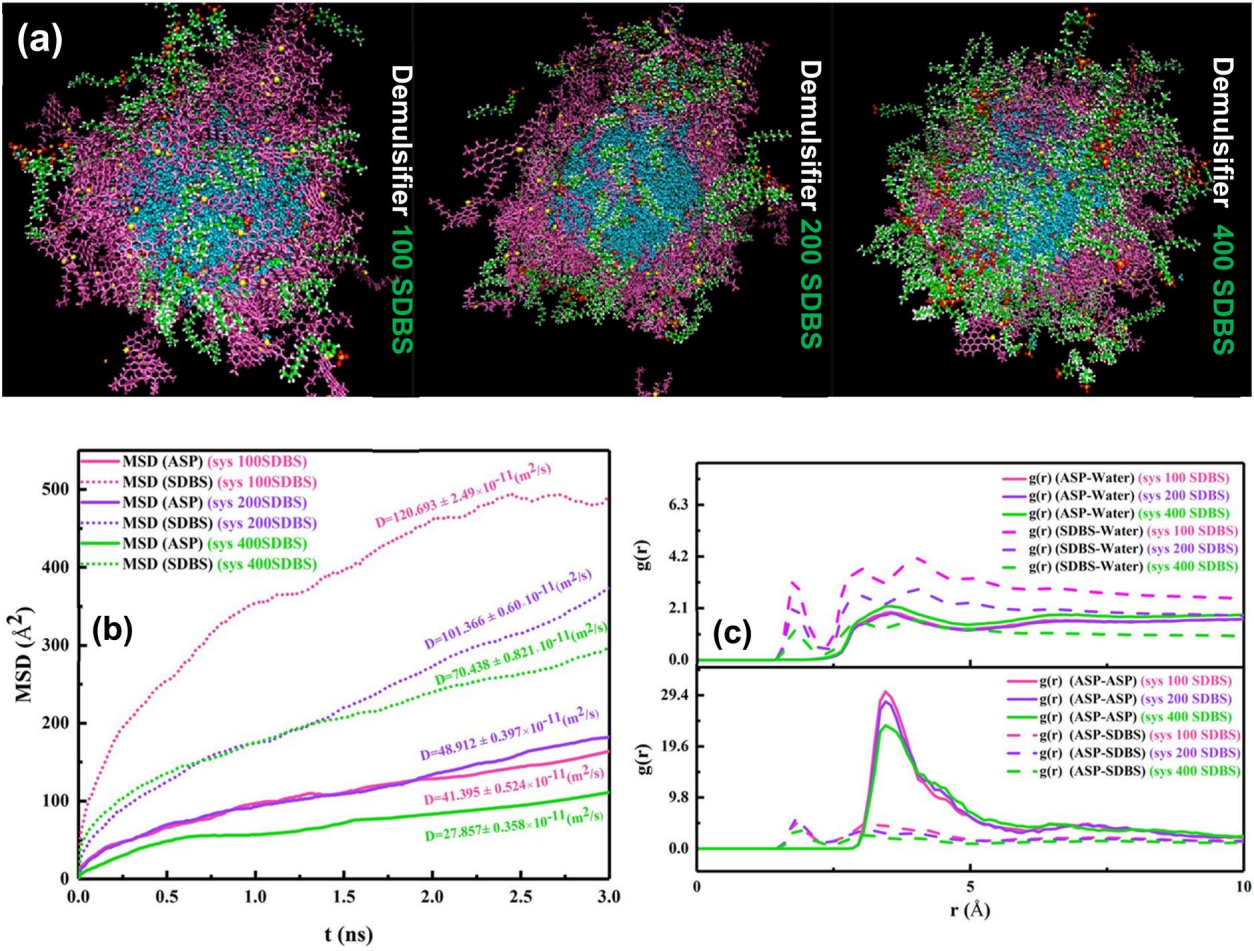
The snapshot of systems with 100, 200, and 400 ASP molecules, respectively (**a**). The mean squared displacement and its slope (diffusion coefficient) belonging to ASP and demulsifier molecules for the systems (**b**). The RDF of the systems with the different number of demulsifier molecules (**c**).

After selecting SDBS as the desired surfactant, the effect of the demulsifier concentration on the interfacial film was investigated. Figure 6a shows the systems consisting of 100, 200, and 400 SDBS surfactant molecules residing around the water droplets surrounded by ASPs. According to the previous section, the MSD value was utilized to scrutinize the alteration in the ASP film. Figure 6b shows that the MSD (ASP) of the system (200 SDBS) is more than that of others, which shows that the ASP film changes more, whereas 100 SDBS molecules are not propellant enough to cause this change.

But conversely, by redoubling the number of surfactants, from 200 to 400 surfactants, the MSD value decreases. It is noticeable that after the specified number of demulsifiers, the interfacial film structure undergoes fewer changes relative to the initial structure. In addition to the MSD plot, Figure S2 shows that the ASP density figures prove the more ruptures caused on the water surface (system 200 SDBS), and conversely, the more stable arrangement of ASP film (system 400 SDBS), which is observed from the less fluctuation of the density profile.

**Figure 7:**
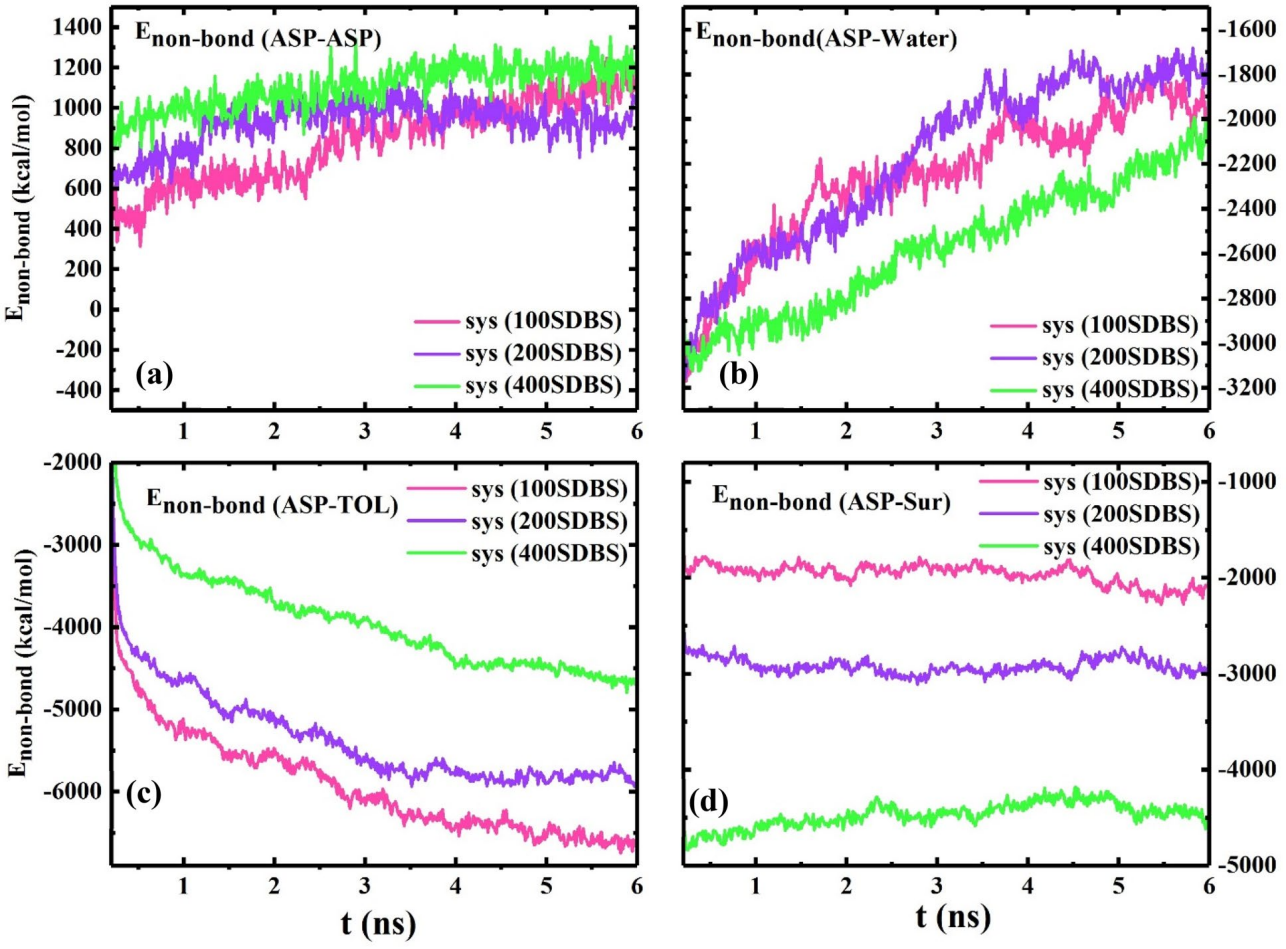
The plot of Enon-bond between some pair contents of the systems with the different number of demulsifier molecules (**a**–**d**).

For the system 400 SDBS, the E_non-bond_ between ASP-ASP molecules reaches a higher level (Fig. 7a), and both g(r)_ASP-ASP_ and g(r)_ASP-SUR_ intensities decline (Fig. 6c), which are acceptable evidence for preserving the ASP film structure by preventing its formation from changing. These promising results can anticipate that beyond the required concentration, at 400 SDBS, the surfactant molecules can join together and form self-aggregation and consequently give rise to retaining the initial arrangement of the ASP film. Therefore, at this concentration, draining the water droplet away^34^ becomes less, and the amount of water extracted from the oil phase will reduce. This prediction is in good agreement with the experimental result in "Bottle test", showing a point in which a reverse trend occurs.

#### The temperature effect on ASP film

**Figure 8:**
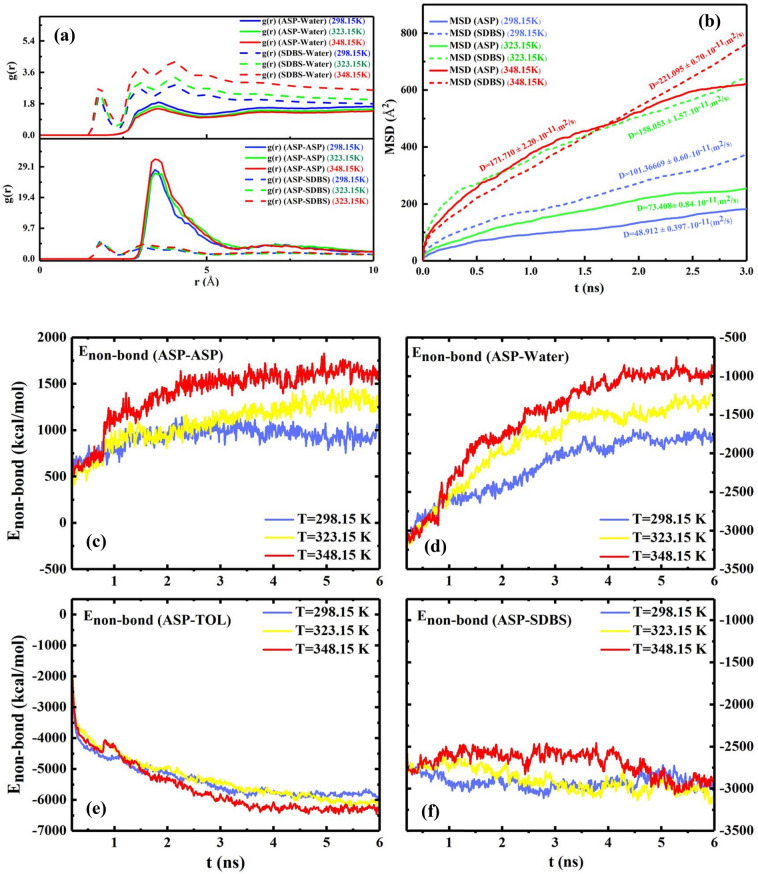
The RDF of the systems with 200 SDBS molecules, as demulsifier, at different temperatures of 298.15 K, 323.15 K, and 348.15 K (**a**). The mean squared displacement and its slope (diffusion coefficient) belonging to ASP and demulsifier molecules at different temperatures (**b**). The plot of ${E}_{non-bond}$ between some pair contents of the systems at different temperatures (**c**–**f**).

The previous section examined the impact of the desired surfactant concentration on the interfacial film. The temperature efficiency on the interfacial film is one unconsidered evaluation in the previous studies of demulsification. In the current part of this study, all system conditions except the temperature are equal. According to the results assessed in both previous parts, some of the determining factors to predict the improvement of the demulsification trend include (a) increasing intensity of g(r) (ASP-ASP), (b) appearing g(r) (ASP-SUR) in less radius, (c) decreasing E_non-bond_ between ASP-ASP molecules, (d) and enhancing the MSD of ASP molecules in the interfacial film. Therefore, it seems unusual that by increasing the temperature, the absolute value of E_non-bond_ among the ASP molecules increases (Fig. 8c–f) while the intensity of g(r)_ASP-ASP_ reaches a higher level (Fig. 8(a)). To investigate the unexpected trend, the results of MSD became more significant. As the MSD plot shows (Fig. 8b), the more the system receives the temperature, the more the ASP displacement occurs. These displacements allow ASP molecules to go through more spatial orientations relative to each other. Mousavi^40^ and coworkers demonstrated the diagonal structure has less distance.

**Figure 9:**
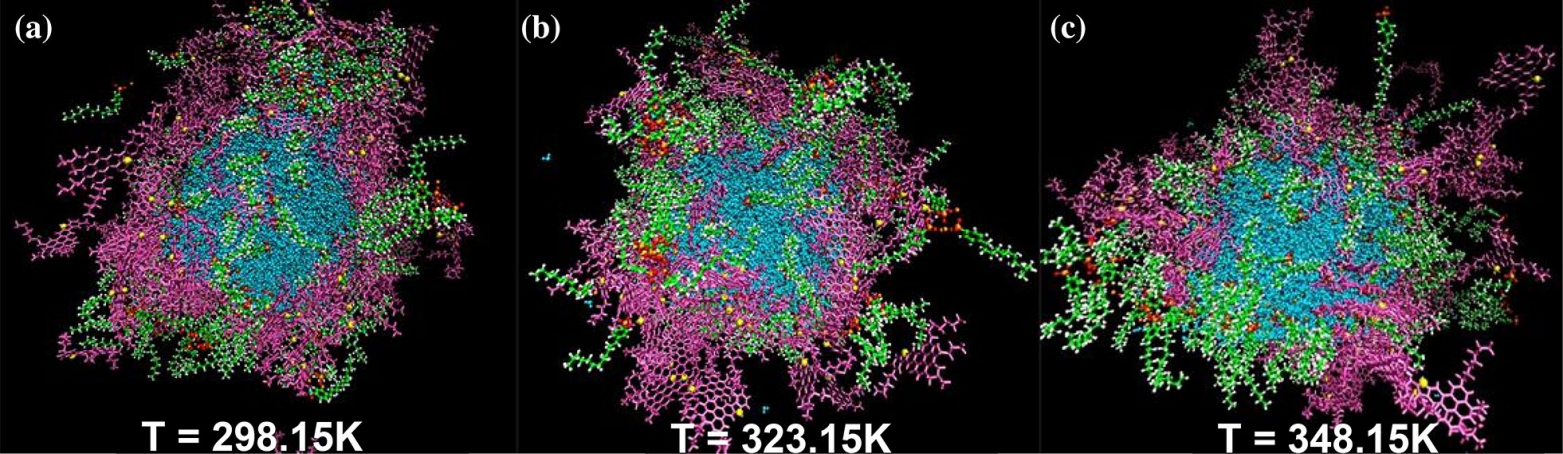
The snapshot of systems with 200 SDBS at different temperatures of 298.15 K (**a**), 323.15 K (**b**), and 348.15 K (**c**).

On the other hand, alleviating the intensity of g(r)_ASP-WAT_ is a piece of evidence of a moderately declining interaction between water and the ASP molecules (Fig. 8a), which causes more conformation changes in the ASP molecules. In addition, the higher value of surfactant's MSD stemmed from increasing temperature conforms to a higher pace of surfactant toward interfacial film, which accelerates the transforming of the interfacial film (Fig. 9). The obtained results advocate that the higher temperature a system has, the more unstable the system becomes. Consequently, it becomes clear that more water would be separated at a higher temperature. The results are consistent with those of experimental investigations explained in "Determining the temperature role", which confirms that more water is extracted from the oil phase.

### Quantum calculation

Several studies have shown that the quantum calculation is an adequate method to evaluate the adsorption of demulsifiers on ASP and to assess different interactions between them. Ren and his team used the quantum calculation to estimate the interaction between graphene oxide and ASP^16^. Also, the DFT method was employed by Flores and coworkers to study the correlation between quantum parameters and the experimental behavior of different demulsifiers^41^.

#### The adsorption of surfactants on ASP

**Figure 10:**
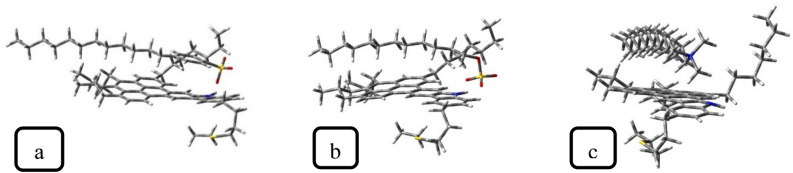
Surfactants adsorption on ASP configuration, (**a**) SDBS, (**b**) SDS (**c**) CTAB.

Table 1 presents the adsorption energy of surfactants on ASP (calculated using Eq. 1). The adsorption energies of anionic surfactants are relatively equal, whereas the adsorption energy of CTAB is notably lower than theirs. This trend was confirmed by E_int_ calculated in the MD simulation ("Effect of demulsifier intrinsic features"). Generally, in the demulsification process, a higher adsorption energy can be more effective because it can be conducive to the transformation of the interfacial ASP film. Moreover, the SDBS adsorption configuration on ASP (Fig. 10) shows that the SDBS benzene ring interacts with the aromatic ring of the ASP structure, which results from the π–π interaction. According to some research investigations^26,42^, the π–π interaction between ASP and the demulsifier has positive effects on the demulsification function. In continuation of this section, some parameters influencing surfactant–ASP interactions will be evaluated.

**Table 1:** The HOMO, LUMO energy, energy gap, hardness, for ASP, surfactants, and complexes (upon eV.).

Compound	$HOMO$	$LUMO$	${E}_{g}$	$\eta$	$\mu$	$-{E}_{ads} (\text{kJ }{\text{mol}}^{-1})$
ASP	− 6.35	− 0.77	5.58	2.79	− 3.56	*
SDBS	− 4.01	3.02	7.03	3.52	− 0.49	*
SDS	− 4.10	3.18	7.28	3.64	− 0.46	*
CTAB	− 10.54	− 2.72	7.82	3.91	− 6.63	*
ASP-SDBS	− 4.16	1.15	5.31	2.65	*	199
ASP-SDS	− 4.24	1.22	5.46	2.73	*	210
ASP-CTAB	− 8.29	− 3.08	5.21	2.60	*	127

Also electronic chemical potential for ASP, surfactants, and complexes (upon eV.). The adsorption energy (kJ/mol).

**Figure 11:**
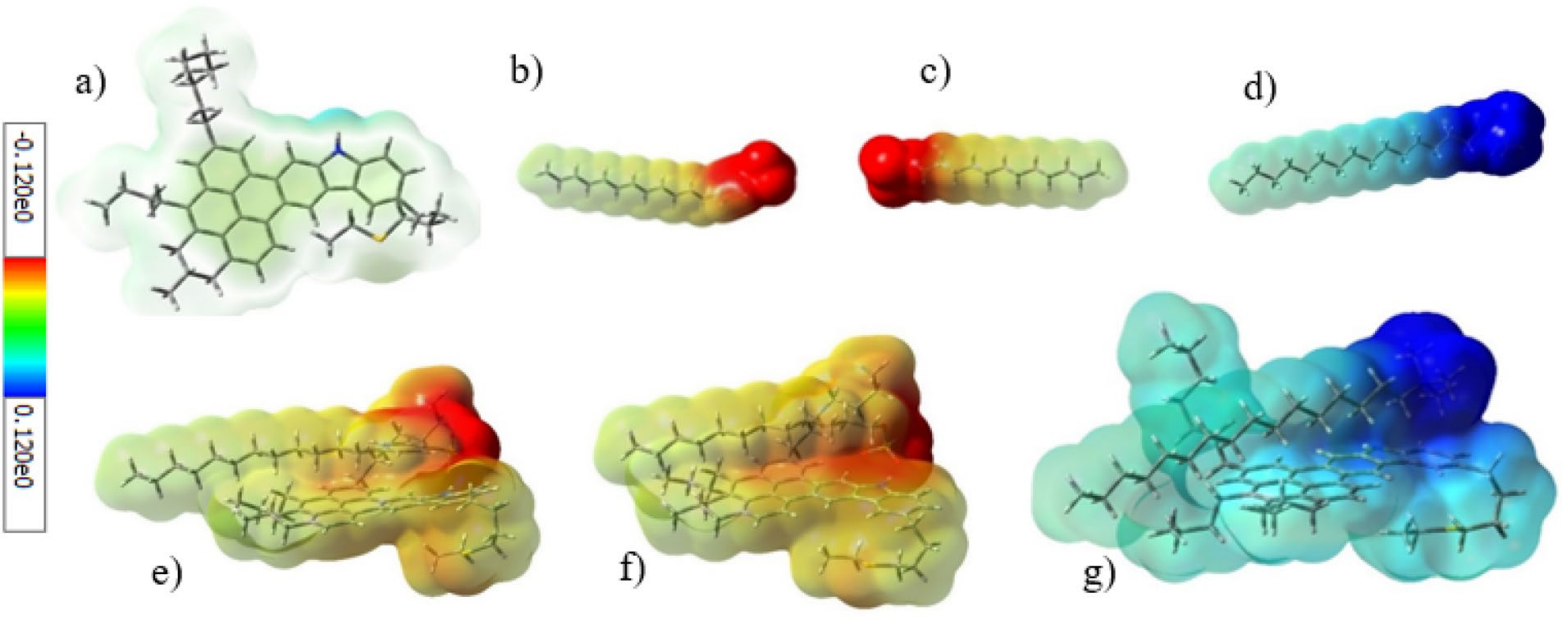
The ESP of (**a**) ASP. (**b**) SDBS, (**c**) SDS, (**d**) CTAB, (**e**) SDBS on ASP, (**f**) SDS on ASP, and (**g**) CTAB on ASP.

An electrostatic interaction, as one of the possible interactions between the surfactant and ASP, is attributed to both the net charge of ionic surfactants and different potential charges of ASP’s heteroatoms and functional groups. To investigate the electrostatic interaction, the electrostatic surface potential (ESP) was used. Figure 11 indicates the ESP for ASP and surfactants, with red and blue colors representing the negative and positive potential, respectively. Therefore, the anionic and cationic surfactants have a negative and positive potential respectively, which is related to the negative and positive charges of their heads. Despite the neutral nature of ASP, both sites with a positive and negative potential can be seen on it simultaneously. It is noticeable that the blue color near the nitrogen atom refers to the existing positive charge, whereas the lack of clarity of the yellow color shows that the negative potential was distributed in many areas of the ASP structure.

The ASP site, containing a positive potential, is an suitable position for the adsorption of anionic surfactants, on which the anionic surfactant heads were adsorbed. In addition, Fig. 11 shows the chnage in the ASP potential area, which stems from the strong interaction caused by the adsorption of the surfactant. Contrary to the anionic surfactants, the CTAB surfactant head, containing a positive potential, cannot be adsorbed on this site because of the electrostatic repulsion. Thus, the electrostatic attraction between ASP and the anionic surfactants, SDBS and SDS, plays a key role in increasing their absolute adsorption energy at a certain distance.

The HOMO and LOMO energies were calculated for some contents including surfactants, ASP, and the complex of an adsorbed surfactant on ASP. The electronic properties of contents can assist us to understand the interaction between them. Figure S6 in SI shows the graphical shape of HOMO and LUMO for ASP, surfactants, and surfactants-ASP. In addition to the shapes of HOMO and LUMO, the energy gap is an important parameter calculated from the HOMO and the LUMO energies. The energy gap of ASP-SDBS is 5.31 eV (Table 1), which is lower than the energy gap of both ASP and SDBS individually. The reduction of the energy gap is an index for the interaction between ASP and SDBS^43^. This phenomenon was observed for two other surfactants.

The global hardness is another parameter derived from the HOMO and LUMO energy, which is an index for reactivity. Thus, a compound with a higher energy gap is harder and less reactive^25^. Between the two anionic surfactants, SDBS with a lower hardness value became more polarized than SDS. So, during the interaction with ASP, SDBS was more effective than SDS. The electronic chemical potential of different compounds, estimated using the HOMO and LUMO energies, can determine the direction of the electron transfer. The ASP molecule has a lower electronic chemical potential than both of the anionic surfactants, so it conducts and transfers electrons towards ASP. This behavior was approved with an increase in the red area of ASP (Fig. 11). On the other hand, the lower chemical potential of CTAB, compared with ASP, leads to a reverse behavior for CTAB.

Several oxygen atoms available in the head group of anionic surfactants enable the formation of hydrogen bonds (HB) between ASP and the anionic surfactants. The results of the AIM analysis reveal that one of the oxygen atoms available in an anionic surfactant structure could form the hydrogen bond with the hydrogen connected to nitrogen in ASP. By comparing the charge density, ρ, and the bond energy of HBs, it can be concluded that the HB strength between ASP and SDS is slightly stronger than the HB strength between ASP and SDBS (Table 2), which is confirmed by a shorter HB length. The HB length of anionic surfactants is consistent whit the g(r) obtained in the MD simulation section (Fig. 4e). Also, it should be emphasized that HB formation between ASP and the demulsifiers can deform the ASP films, thereby accelerating the demulsification process. According to these promising results, it is expected that both anionic surfactants would separate water more efficiently than CTAB.

**Table 2:** The values of electron density (ρ), its Laplacian (∇2ρ), the energy of hydrogen bonding $\left({E}_{HB}\right),$ bond length, and different charge (D.C) for hydrogen bonding between ASP surfactants.

Compounds	Atoms	$\rho$	${\nabla }^{2}\rho$	${E}_{HB} (\text{kj }{\text{mol}}^{-1})$	$r (\text{nm})$	$D.$C
ASP-SDBS	$S-O\dots H-N$	0.036	0.032	42	0.18	1.53
ASP-SDS	$S-O\dots H-N$	0.038	0.035	48	0.17	1.52

### Experimental section

#### Bottle test

The size of water droplets in the emulsion was estimated to be 2.3 ± 1.5 μm by optical microscope. Several studies have shown that the size of water droplets in the water-in-oil emulsion can vary from a sub-micrometer^44^ to a several micrometers^44^.

**Figure 12:**
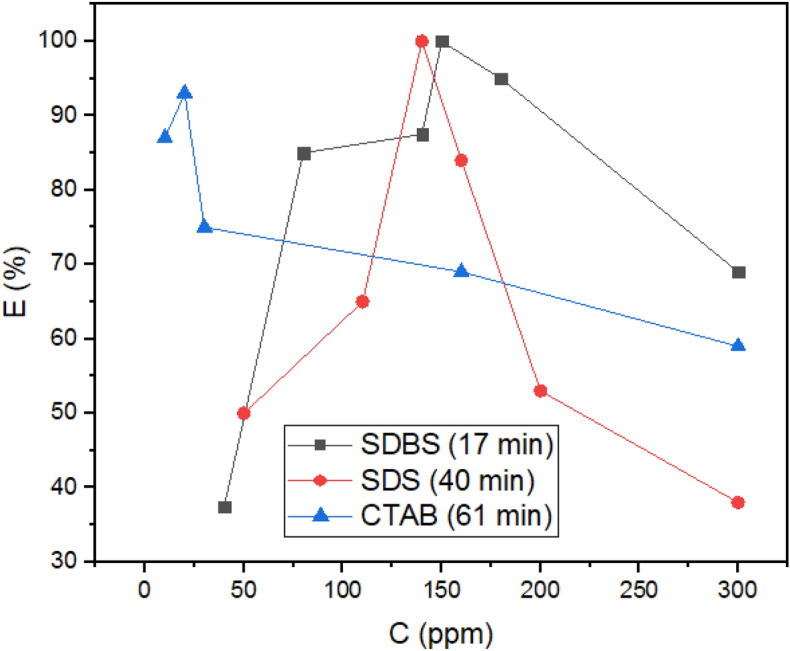
The demulsification performance of SDBS, SDS and CTAB at 298 K.

The results of the bottle test for all surfactants (Fig. 12) indicate that the anionic surfactants as demulsifiers are more efficient than the cationic surfactant (CTAB). According to the figures, SDBS and SDS completely removed water from crude oil (at 17 and 40 min respectively), whereas CTAB cannot completely dehydrate it (95% at 61 min). The amount of water remaining in crude oil after demulsification by surfactant CTAB is more than allowed^45^. This trend was anticipated with MD and quantum calculations. In this way, that anionic surfactants have stronger interactions with ASP (Table 1; Fig. 4b). The interaction of demulsifiers with ASP changes the ASP hydrophilicity^46^, thereby coagulating the water droplets more rapidly. The formation of HB for anionic surfactants, as another factor calculated with AIM, propels the process of demulsification^26^. Also, hydrogen bonding can affect asphaltene accumulation^47^. It should be pointed out that all surfactants applied in this study can dehydrate crude oil by rupturing the ASP film surrounding the water droplets. The changes in E_int_ and g(r) related to ASP-ASP, which were computed as evidence of film changes in the MD section, took place following the addition of demulsifiers to crude oil. As shown in Fig. 1, SDBS has a benzene ring that facilitates its diffusion in the crude oil medium^26^, thus enhancing the SDBS function in comparison to SDS. Besides, the SDBS benzene ring interacts with ASP available in the interfacial film^16^ and helps to coalesce the individual water droplets. These observations confirm the critical role of the benzene ring in the demulsification process, which is consitent with some approving research about the constructive effect of the aromatic ring^16,26,42^.The efficiency of demulsification can be manipulated by another parameter known as hydrophilic–lipophilic balance (HLB). The higher the HLB value the surfactant has, the more the surfactant is hydrophilic. The HLB value for SDBS and SDS were reported to be 10.6 and 40, respectively^48^. The SDS head with a higher value of HLB has more tendency to be entrapped in the water phase^49^, whereas the moderate HLB value for SDBS helps to maintain its head on the water–crude oil interface, so it would improve the demulsification function. Various factors, which belong to the demulsifier nature, affect the demulsification efficiency, but many papers have reported that the demulsifiers with moderate hydrophilicity function better^49,50^.

As mentioned in the MD part (Fig. 6a), the sealing of ruptures by the extra surfactants takes place after an optimum concentration, so it decreased the dehydration efficiency of the three surfactants. Electrostatic repulsion can have a great influence on the behavior of systems containing ionic surfactants^51^, therefore the electrostatic repulsion of the ionic surfactant at the interface is another reason^52^. Based on the MD results indicating a greater number of CTAB sealed available ruptures on the interface (Fig. 5b), it is expected that CTAB as a demulsifier would have a less optimum concentration. Besides, SDS and SDBS with a lower and similar number of adsorbed demulsifiers on ruptures should have an equal optimum concentration, i.e., higher than the CTAB optimum concentration. All predictions about the optimum concentration were confirmed by the experimental approach, i.e., the optimum concentrations for CTAB, SDS and SDBS were 20, 140, and 150 ppm, respectively (Fig. 12). The decrease in the dehydration efficiency after the optimum concentration has been reported in some studies^26,53,54^.

Figure 12 indicates that beyond the specified concentration which belong to all of the intended demulsifiers, the separated water decreses. There are two reasonsfor the phenomenon mentioned above, namely (1) the electrostatic repulsions between identical demulsifiers, and (2) the sealing film of the demulsifier surrounding the ASP interfacial film. These phenomena are common in the demulsification process and have been reported in some studies^26,55^.

#### Determining the temperature role

**Figure 13:**
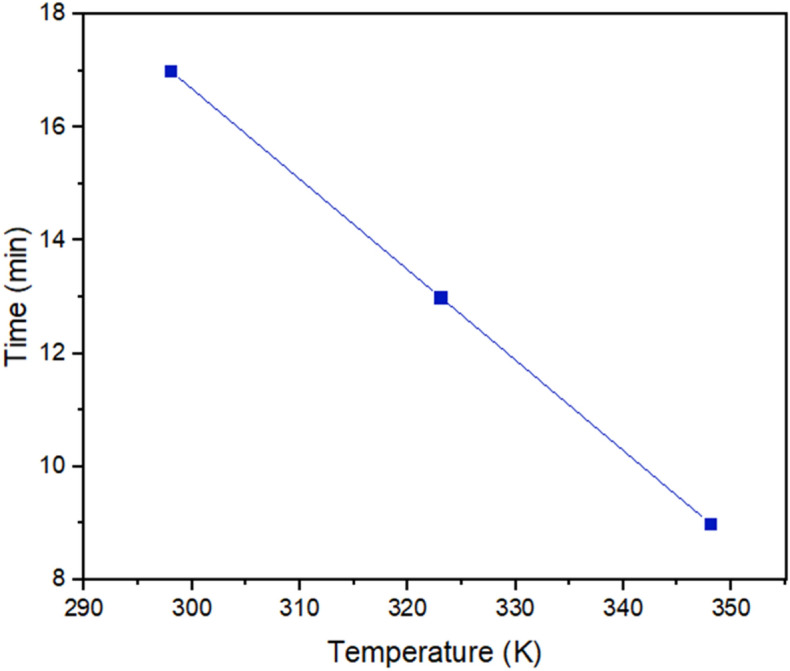
The temperature effect on demulsification time for the optimum concentration of SDBS at 298, 323, and 348 K.

To study how temperature affects the demulsification process, the SDBS optimum concentration was regarded as the desired concentration, and the temperature range was considered 298, 323, and 348 K (Fig. 13). Some studies have demonstrated the importance of the temperature effect on the demulsification process^26,56,57^. The bottle test results explained that the demulsification time for the complete removal of water from crude oil declined by increasing the temperature. The more the temperature of the system is, the better the demulsification process function would be. It is time to scrutinize the underlying factors behind the temperature as a propellant factor. The reduction in viscosity caused by increasing the temperature is known as a significant factor in the improvement of demulsification performance^58^ because in this situation, water droplets could diffuse together easily. Besides, increasing the temperature not only accelerates the movement of demulsifiers towards the interface^26^, but also can increase the kinetic energy of water droplets and the collision between them^59^. The MSD results displayed in Fig. 8b confirm the increase in movement of the the demulsifier along with the increase in the temperature.

The stability of the ASP film around the water droplet can change with temperature changes^60^, the MD results mentioned above predicted that the displacement of ASP increases with the increase in temperature. So, it is more likely to alter the interface film and extract the water efficiently. In this way, the water droplets coalesce quickly. This trend was confirmed by the bottle test in this section.

## Conclusion

This work provides useful insights into how the interfacial film, as an obstacle to demulsification, changes during the demulsification process. So, it should be noticed that the transformation of the interfacial film is a predominant step in rupturing the interfacial film and draining the water droplets into each other. Moreover, the factors which transform the film have been carefully scrutinized. Therefore, the MD simulation and quantum mechanics studies show that it is imperative to keep a demulsifier molecule in the proximity of ASP. This is attributed to the electrostatic interaction and hydrogen bonding formed between ASP and demulsifier molecules. MD simulation demonstrated that a suitable demulsifier has a tendency not only to ASP molecules but also to the water molecule, both of which contribute to the film alteration. Next, by increasing the concentration of demulsifier molecules, they were conducted towards the film ruptures and encapsulated the water droplets more. The consequence of this effect was observed in the decline in the demulsification performance in the experimental section. Finally, at a higher temperature, raising the diffusion coefficient of the demulsifier as well as the possibility of ASP movement can accelerate the interfacial film transition.

### Supplementary Information

Supplementary Information.

## Data Availability

The datasets used and/or analysed during the current study available from the corresponding author on reasonable request.

## References

[1] Azizi N, Bashipour F (2022). Demulsification of water-in-oil emulsions applying Fe_3_O_4_ magnetic nanoparticles for demulsifier modification: Experimental optimization via response surface methodology. J. Pet. Sci. Eng..

[2] Duan M (2017). Layer-by-layer assembled film of asphaltenes/polyacrylamide and its stability of water-in-oil emulsions: A combined experimental and simulation study. J. Phys. Chem. C.

[3] Nikolov A, Wasan D (2021). Methods to monitor water-in-oil film thinning and stability: An application to bitumen demulsification. J. Colloid Interface Sci..

[4] Liu J, Zhao Y, Ren S (2015). Molecular dynamics simulation of self-aggregation of asphaltenes at an oil/water interface: Formation and destruction of the asphaltene protective film. Energy Fuels.

[5] Alvarez F (2011). Dissipative particle dynamics (DPD) study of crude oil-water emulsions in the presence of a functionalized co-polymer. Energy Fuels.

[6] Zhang LY, Xu Z, Masliyah JH (2003). Langmuir and Langmuir–Blodgett films of mixed asphaltene and a demulsifier. Langmuir.

[7] Headen TF, Boek ES, Jackson G, Totton TS, Müller EA (2017). Simulation of asphaltene aggregation through molecular dynamics: Insights and limitations. Energy Fuels.

[8] Kilpatrick PK (2012). Water-in-crude oil emulsion stabilization: Review and unanswered questions. Energy Fuels.

[9] Gao F, Xu Z, Liu G, Yuan S (2014). Molecular dynamics simulation: The behavior of asphaltene in crude oil and at the oil/water interface. Energy Fuels.

[10] Biniaz P, Farsi M, Rahimpour MR (2016). Demulsification of water in oil emulsion using ionic liquids: Statistical modeling and optimization. Fuel.

[11] Atta AM, Abdullah MMS, Al-Lohedan HA, Ezzat AO (2018). Demulsification of heavy crude oil using new nonionic cardanol surfactants. J. Mol. Liq..

[12] Fuentes JV (2021). Alkylacrylic-carboxyalkylacrylic random bipolymers as demulsifiers for heavy crude oils. Sep. Purif. Technol..

[13] Zhang L, Greenfield ML (2007). Molecular orientation in model asphalts using molecular simulation. Energy Fuels.

[14] Kuznicki T, Masliyah JH, Bhattacharjee S (2008). Molecular dynamics study of model molecules resembling asphaltene-like structures in aqueous organic solvent systems. Energy Fuels.

[15] Ahmadi M, Chen Z (2020). Insight into the interfacial behavior of surfactants and asphaltenes: Molecular dynamics simulation study. Energy Fuels.

[16] Liu J (2017). Recyclable magnetic graphene oxide for rapid and efficient demulsification of crude oil-in-water emulsion. Fuel.

[17] Martínez JM, Martínez L (2003). Packing optimization for automated generation of complex system’s initial configurations for molecular dynamics and docking. J. Comput. Chem..

[18] Tirjoo A, Bayati B, Rezaei H, Rahmati M (2019). Molecular dynamics simulations of asphaltene aggregation under different conditions. J. Pet. Sci. Eng..

[19] Mahoney MW, Jorgensen WL (2000). A five-site model for liquid water and the reproduction of the density anomaly by rigid, nonpolarizable potential functions. J. Chem. Phys..

[20] Javadian S, Khosravian M (2018). Revealing factors governing self-assembly morphology of fatty acid on graphene synthesized by surfactant-assisted LPE: A joint MD, SAPT (DFT), and experimental study. J. Phys. Chem. C.

[21] Vanommeslaeghe K (2010). CHARMM general force field: A force field for drug-like molecules compatible with the CHARMM all-atom additive biological force fields. J. Comput. Chem..

[22] Lin S, Blankschtein D (2010). Role of the bile salt surfactant sodium cholate in enhancing the aqueous dispersion stability of single-walled carbon nanotubes: A molecular dynamics simulation study. J. Phys. Chem. B.

[23] York DM, Darden TA, Pedersen LG (1993). The effect of long-range electrostatic interactions in simulations of macromolecular crystals: A comparison of the Ewald and truncated list methods. J. Chem. Phys..

[24] Javadian S, Ektefa F (2015). An efficient approach to explore the adsorption of benzene and phenol on nanostructured catalysts: A DFT analysis. RSC Adv..

[25] Javadian S (2021). Graphene quantum dots based magnetic nanoparticles as a promising delivery system for controlled doxorubicin release. J. Mol. Liq..

[26] Javadian S, Sadrpoor SM (2020). Demulsification of water in oil emulsion by surface modified SiO_2_ nanoparticle. J. Pet. Sci. Eng..

[27] Li Z (2021). Study on demulsifier crude oil interactions at oil-water interface for crude oil dehydration. Colloids Surf. A Physicochem. Eng. Asp..

[28] Hong X (2022). Molecular understanding on migration and recovery of shale gas/oil mixture through a pore throat. Energy Fuels.

[29] Cadena-Nava RD, Consultchi A, Ruiz-Garcia J (2007). Asphaltene behavior at interfaces. Energy Fuels.

[30] Babazadeh H, Foroutan M (2019). Investigation of the surfactant effects on oil-water separation on nano-crystalline titanium dioxide substrate using molecular dynamics simulation. Appl. Surf. Sci..

[31] Giorgino T (2014). Computing 1-D atomic densities in macromolecular simulations: The density profile tool for VMD. Comput. Phys. Commun..

[32] Motaee A, Javadian S, Khosravian M (2021). Influence of adsorption energy in graphene production via surfactant-assisted exfoliation of graphite: A graphene-dispersant design. ACS Appl. Nano Mater..

[33] Hong X (2022). Competitive adsorption of asphaltene and n-heptane on quartz surfaces and its effect on crude oil transport through nanopores. J. Mol. Liq..

[34] Nikolov AD, Wasan DT (1997). Effects of film size and micellar polydispersity on film stratification. Colloids Surf. A Physicochem. Eng. Asp..

[35] Headen TF, Boek ES, Skipper NT (2009). Evidence for asphaltene nanoaggregation in toluene and heptane from molecular dynamics simulations. Energy Fuels.

[36] Yi H (2018). Surface wettability of montmorillonite (0 0 1) surface as affected by surface charge and exchangeable cations: A molecular dynamic study. Appl. Surf. Sci..

[37] Costa JLLFS, Simionesie D, Zhang ZJ, Mulheran PA (2016). Aggregation of model asphaltenes: A molecular dynamics study. J. Phys. Condens. Matter.

[38] Li N (2022). Deformation and breakup mechanism of water droplet in acidic crude oil emulsion under uniform electric field: A molecular dynamics study. Colloids Surf. A Physicochem. Eng. Asp..

[39] Zhang M, Mao H, Jin Z (2021). Molecular dynamic study on structural and dynamic properties of water, counter-ions and polyethylene glycols in Na-montmorillonite interlayers. Appl. Surf. Sci..

[40] Mousavi M, Abdollahi T, Pahlavan F, Fini EH (2016). The influence of asphaltene-resin molecular interactions on the colloidal stability of crude oil. Fuel.

[41] Flores CA (2014). Anion and cation effects of ionic liquids and ammonium salts evaluated as dehydrating agents for super-heavy crude oil: Experimental and theoretical points of view. J. Mol. Liq..

[42] Kang W (2018). ​ Demulsification performance, behavior and mechanism of different demulsifiers on the light crude oil emulsions. Colloids Surf. A Physicochem. Eng. Asp..

[43] Vatanparast M, Shariatinia Z (2019). Hexagonal boron nitride nanosheet as novel drug delivery system for anticancer drugs: Insights from DFT calculations and molecular dynamics simulations. J. Mol. Graph. Model..

[44] MaiaFilho DC, Ramalho JBVS, Spinelli LS, Lucas EF (2012). Aging of water-in-crude oil emulsions: Effect on water content, droplet size distribution, dynamic viscosity and stability. Colloids Surf. A Physicochem. Eng. Asp..

[45] Yonguep E, Fabrice KK, Katende JK, Chowdhury M (2022). Formation, stabilization and chemical demulsification of crude oil-in-water emulsions: A review. Pet. Res..

[46] O’Neil B, Maley D, Lalchan C (2015). Prevention of acid-induced asphaltene precipitation: A comparison of anionic vs cationic surfactants. J. Can. Pet. Technol..

[47] El-Nagar RA, Nessim MI, Ismail DA, Mohamed MG, Ghanem A (2023). Investigation the effect of different ionic liquids based-aryl imidazole on the onset precipitation of asphaltene. Sci. Rep..

[48] Pour AN, Housaindokht MR, Shahri SMK, Babakhani EG, Irani M (2011). Size dependence on reduction kinetic of iron based Fischer–Tropsch catalyst. J. Ind. Eng. Chem..

[49] Yegya Raman AK, Aichele CP (2018). Demulsification of surfactant-stabilized water-in-oil (cyclohexane) emulsions using silica nanoparticles. Energy Fuels.

[50] Huang X (2016). Demulsification of a new magnetically responsive bacterial demulsifier for water-in-oil emulsions. Energy Fuels.

[51] Gharibi H, Sohrabi B, Javadian S, Hashemianzadeh M (2004). Study of the electrostatic and steric contributions to the free energy of ionic/nonionic mixed micellization. Colloids Surf. A Physicochem. Eng. Asp..

[52] Sun T (2002). Influence of demulsifiers of different structures on interfacial dilational properties of an oil–water interface containing surface-active fractions from crude oil. J. Colloid Interface Sci..

[53] Javadian S, Bahri M, Sadrpoor SM, Rezaei Z, Kakemam J (2022). Structure effect in the demulsification performance of cationic surfactants. J. Pet. Sci. Eng..

[54] Hazrati N, Beigi AAM, Abdouss M (2018). Demulsification of water in crude oil emulsion using long chain imidazolium ionic liquids and optimization of parameters. Fuel.

[55] Liu M (2019). The effect of demulsifier on the stability of liquid droplets: A study of micro-force balance. J. Mol. Liq..

[56] Javadian S, Khalilifard M, Sadrpoor SM (2019). Functionalized graphene oxide with core-shell of Fe3O4@ oliec acid nanospheres as a recyclable demulsifier for effective removal of emulsified oil from oily wastewater. J. Water Process Eng..

[57] Husain A (2023). Demulsification of asphaltene stabilized crude oil emulsions by biodegradable ethylcellulose polymers with varying viscosities. Sci. Rep..

[58] Ali N (2015). Novel Janus magnetic micro particle synthesis and its applications as a demulsifier for breaking heavy crude oil and water emulsion. Fuel.

[59] Fang S (2017). An innovative method to introduce magnetism into demulsifier. Chem. Eng. J..

[60] Zolfaghari R, Fakhru’l-Razi A, Abdullah LC, Elnashaie SSEH, Pendashteh A (2016). Demulsification techniques of water-in-oil and oil-in-water emulsions in petroleum industry. Sep. Purif. Technol..

