# Supplementary material for: Taking a look accurately at the alteration of interfacial asphaltene film exposed to the ionic surfactants as demulsifiers
Source: Sci Rep. 2023 Aug 8;13:12837. doi: 10.1038/s41598-023-39731-0 (PMC10409716; doi:10.1038/s41598-023-39731-0)
Supplement: Supplementary file 1 — Supplementary Information. [file 41598_2023_39731_MOESM1_ESM.docx]

Taking a look accurately at the alteration of interfacial asphaltene film exposed to the ionic surfactants as demulsifiers

Soheila Javadian*, S. Morteza Sadrpoor, Mahnaz Khosravian*

Department of Physical Chemistry, Faculty of Basic Science, Tarbiat Modares University,

P.O. Box 14115-175Tehran, I.R. of Iran

Corresponding authors email address: [Javadian_s@modares.ac.ir](mailto:Javadian_s@modares.ac.ir), mahnaz.khosravian@gmail.com

To confirm the force field and method utilized to carry out this study, MSD and self-diffusion coefficient (D) of toluene were calculated. The initial condition includes 150 toluene molecules embedded in the box with dimensions 45×45×45 Å^3^. The Calculation was conducted with an NPT ensemble in T=300 K during 1ns. After terminating the calculation, the box dimensions were reduced to 30.15×30.15×30.15 Å3. According to Figure S1, D_Toluene_ is 2.27×10^-9^m^2^/s, which is equal to the experimental value ^1^.


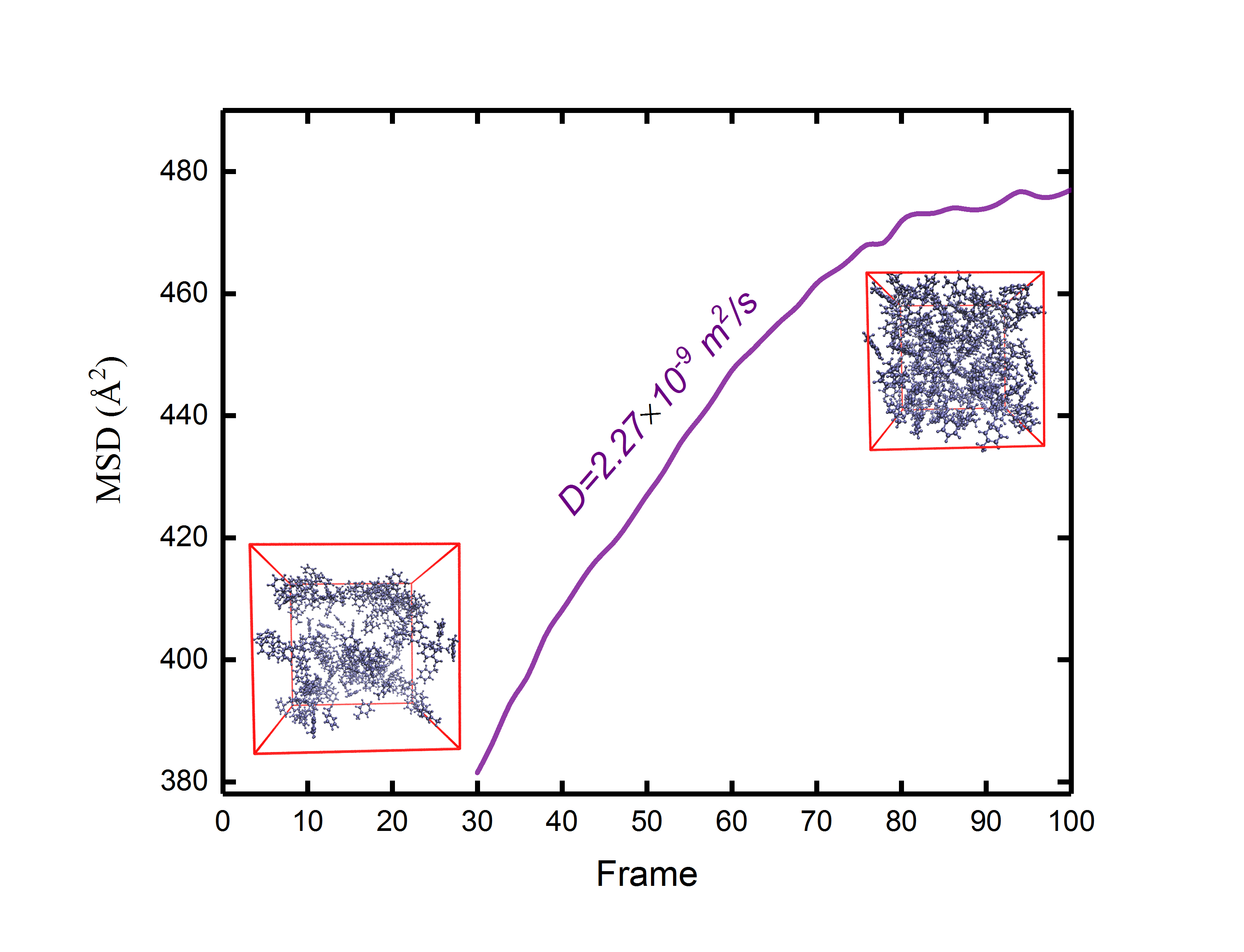


**Figure S1.** The snapshot of initial and final configuration of system containing toluene molecules and their mean squared displacement and its slope (self-diffusion coefficient).


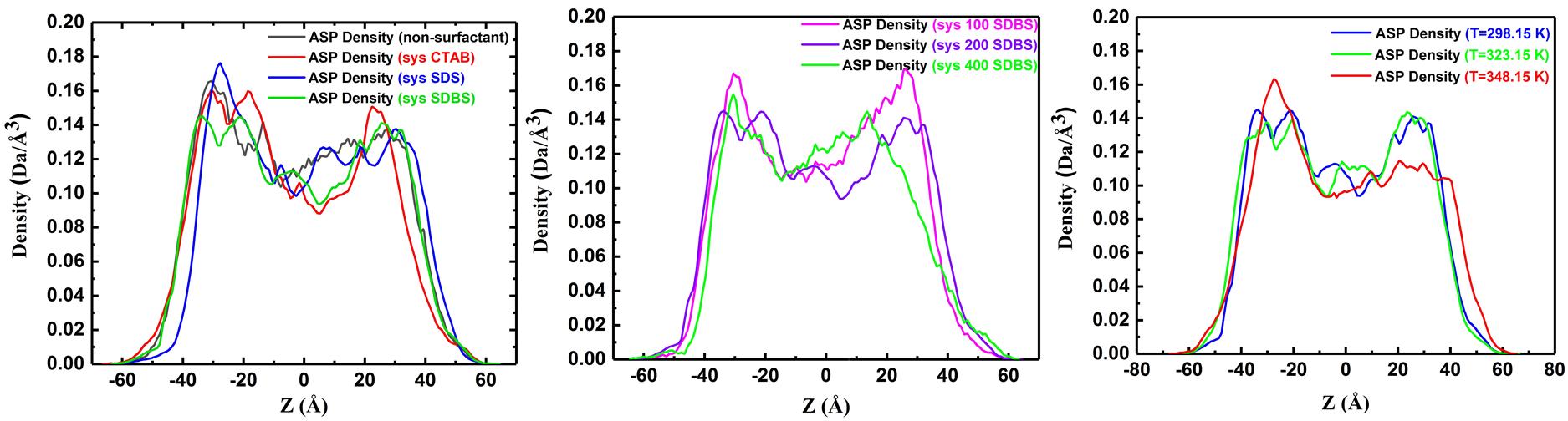


(a)

(c)

(b)

**Figure S2.** The density profile of ASP under different circumstances investigated in this study. Effect of demulsifier intrinsic features (a), the trend of increasing the demulsifier concentration (b), temperature effect (c).

E_non-bond_ discussed in this paper comprises two terms; E_VDW_ and E_ele_ ^2^. The contribution of each term for all conditions, have been exhibited in Figures S3, S4 and S5. These figures are compatible with Enon-bond in Figures 4((a), (b), (c), (d)), 7((a), (b), (c), (d)), and 8((a), (b), (c), (d)). The changes trend related to E_VDW_ and E_ele_ for (ASP-ASP), (ASP-TOL), and (ASP-WAT) demonstrate that between non-ionic content, EVDW plays a key role to determine the variation of E_non-bond_, while in case of E_non-bond_ (ASP-SUR) determining factor for the variation is E_ele_.


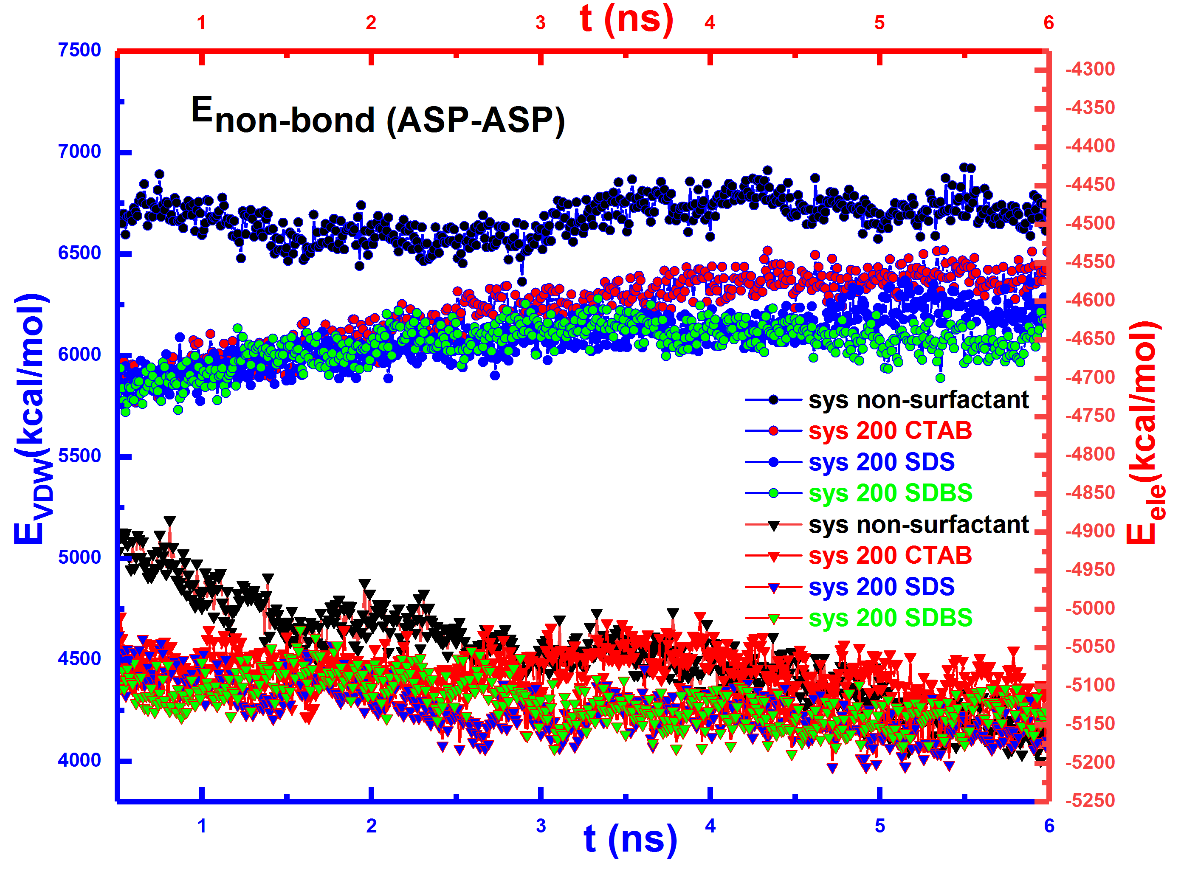

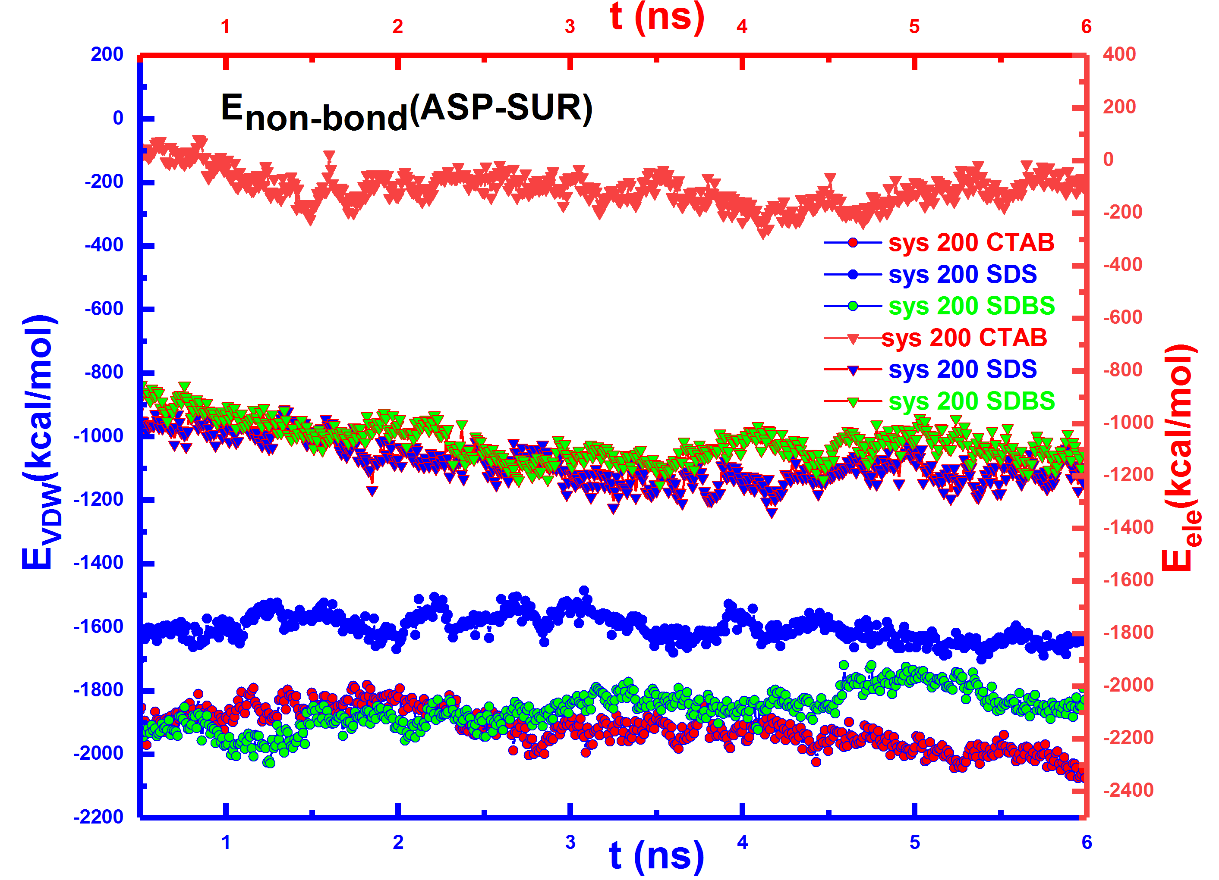


**(a)**

**(b)**


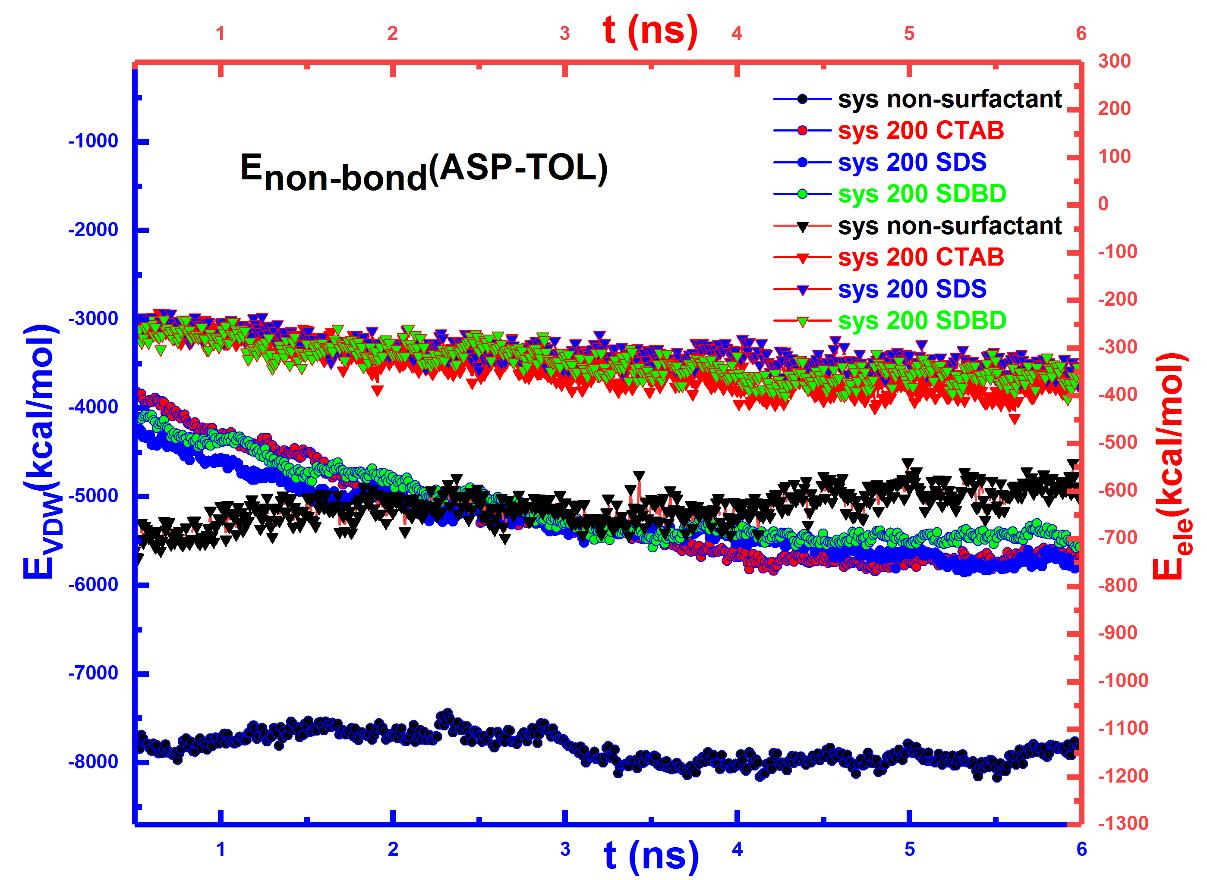

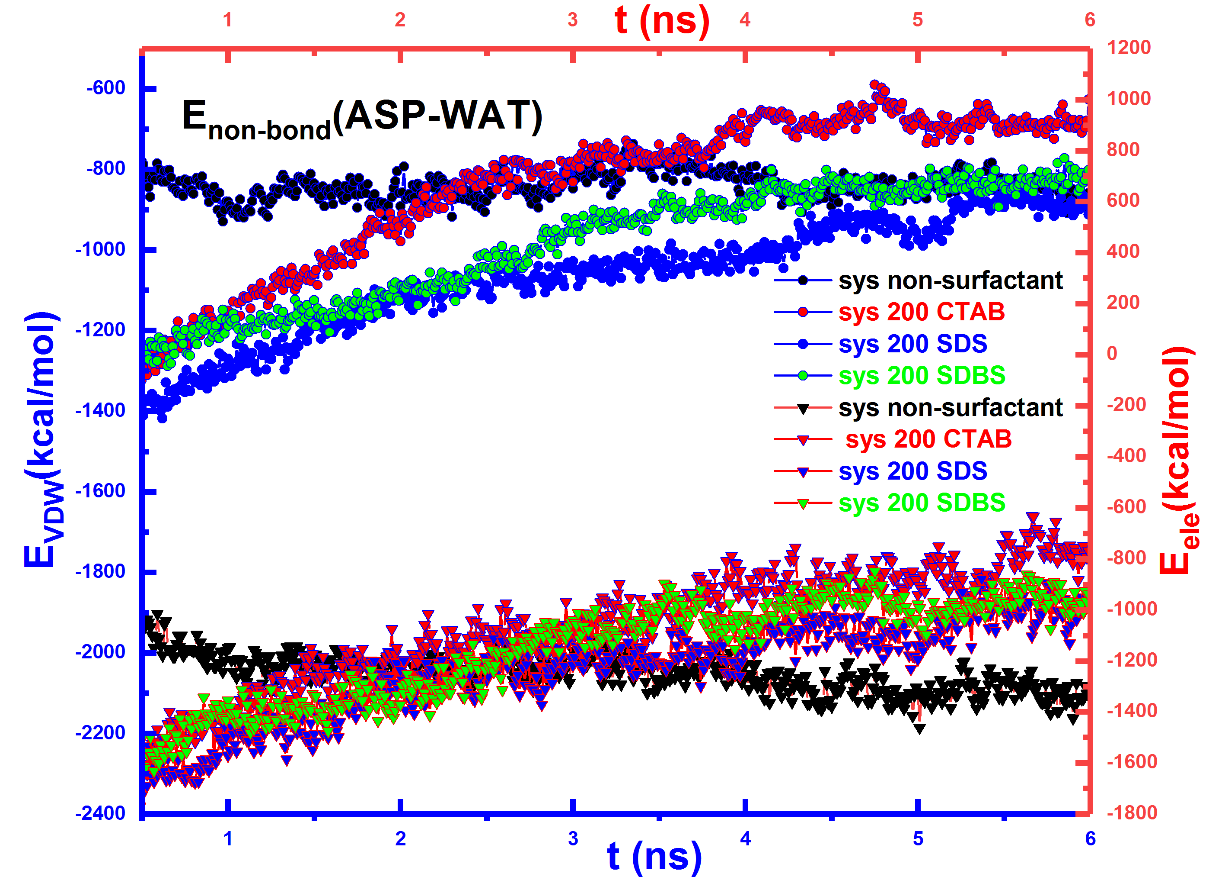


**(c)**

**(d)**

Figure S3. The plot of E_VDW_ and E_ele_ associated with some pair contents of the four systems with non-surfactant, CTAB, SDS, SDBS as demulsifier (a,b,c,d).


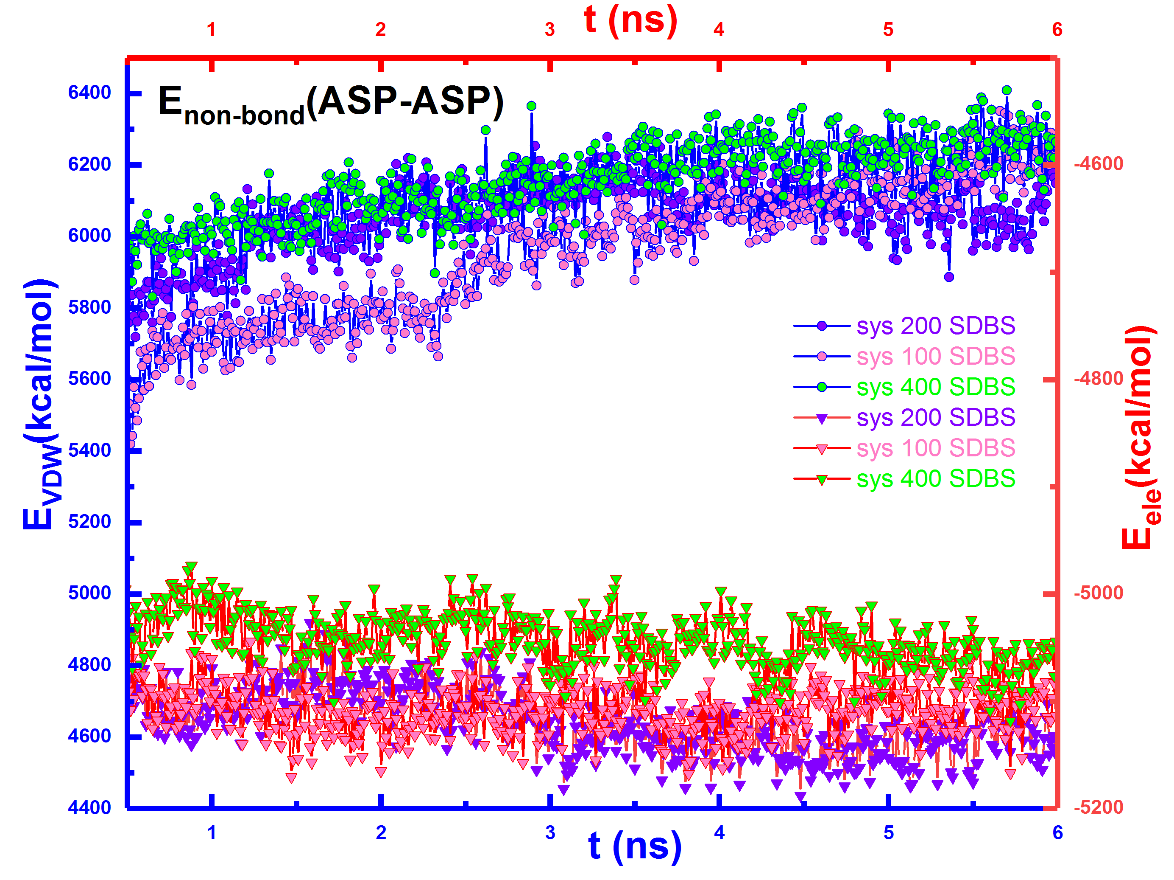

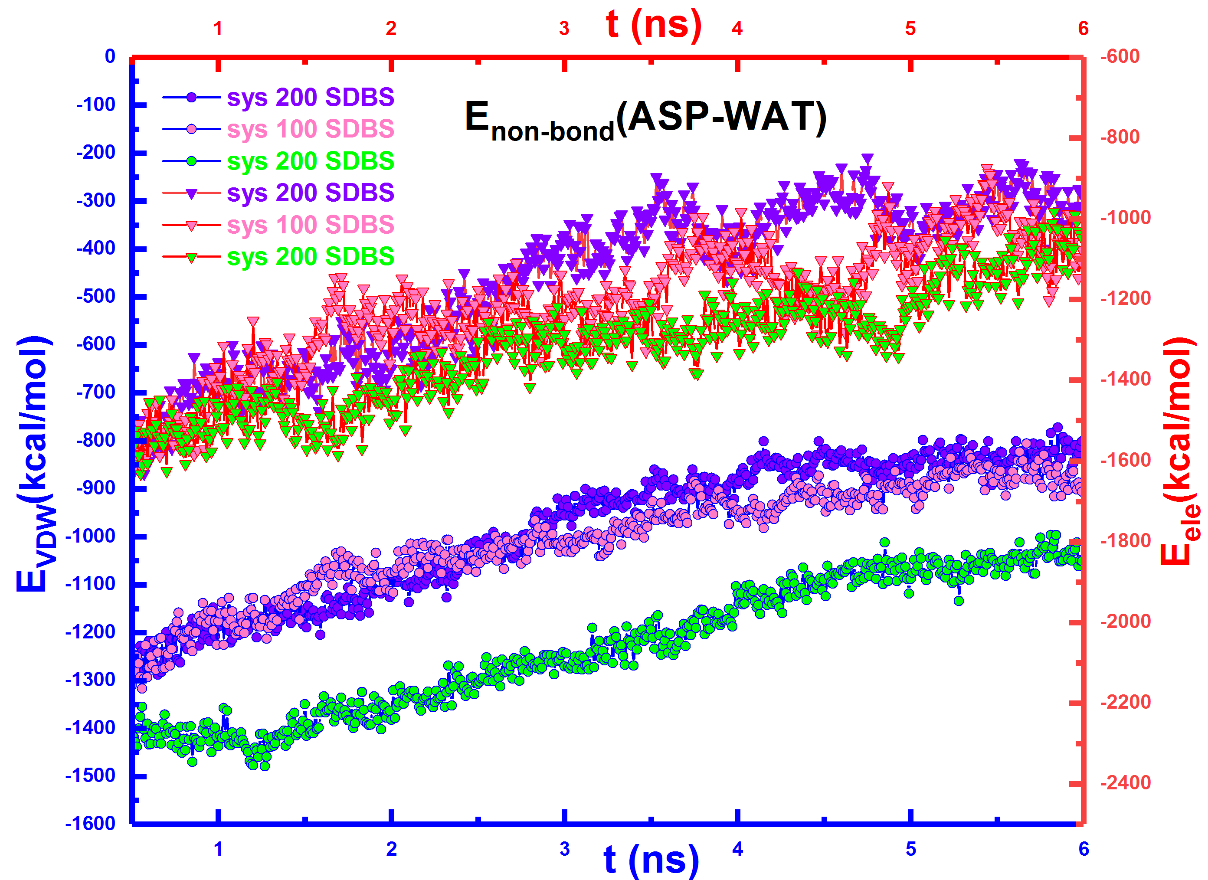


**(a)**

**(b)**


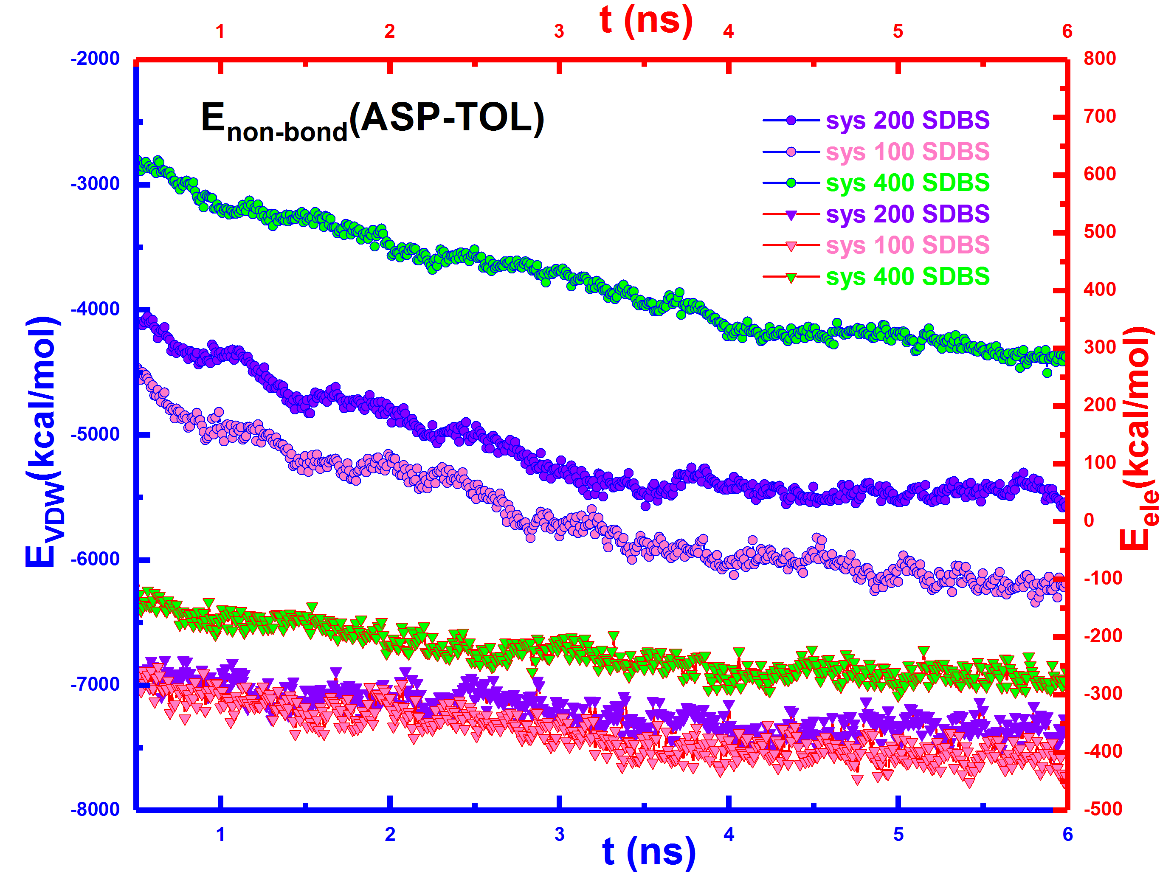

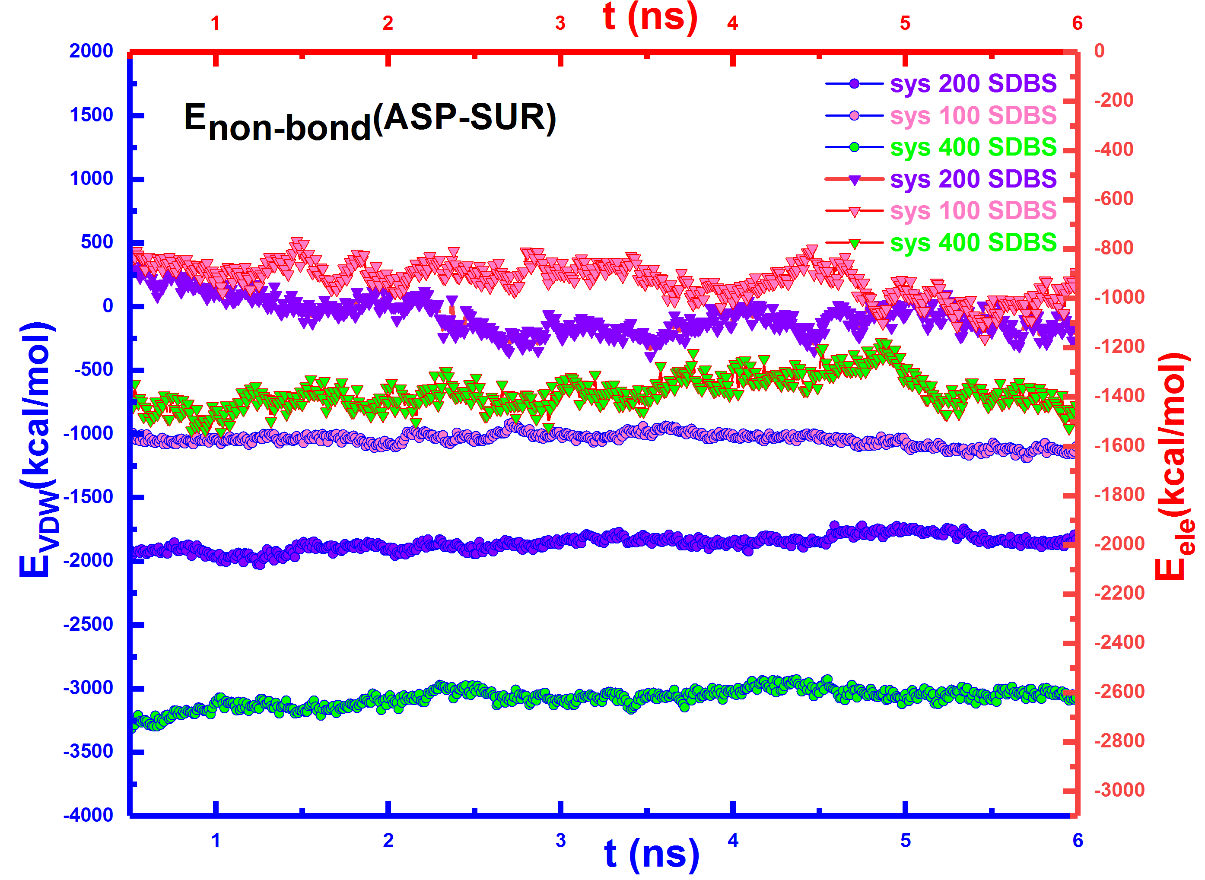


**(c)**

**(d)**

Figure S4. The plot of E_VDW_ and E_ele_ associated with some pair contents of the systems with different number of demulsifier molecules (a, b, c, d).


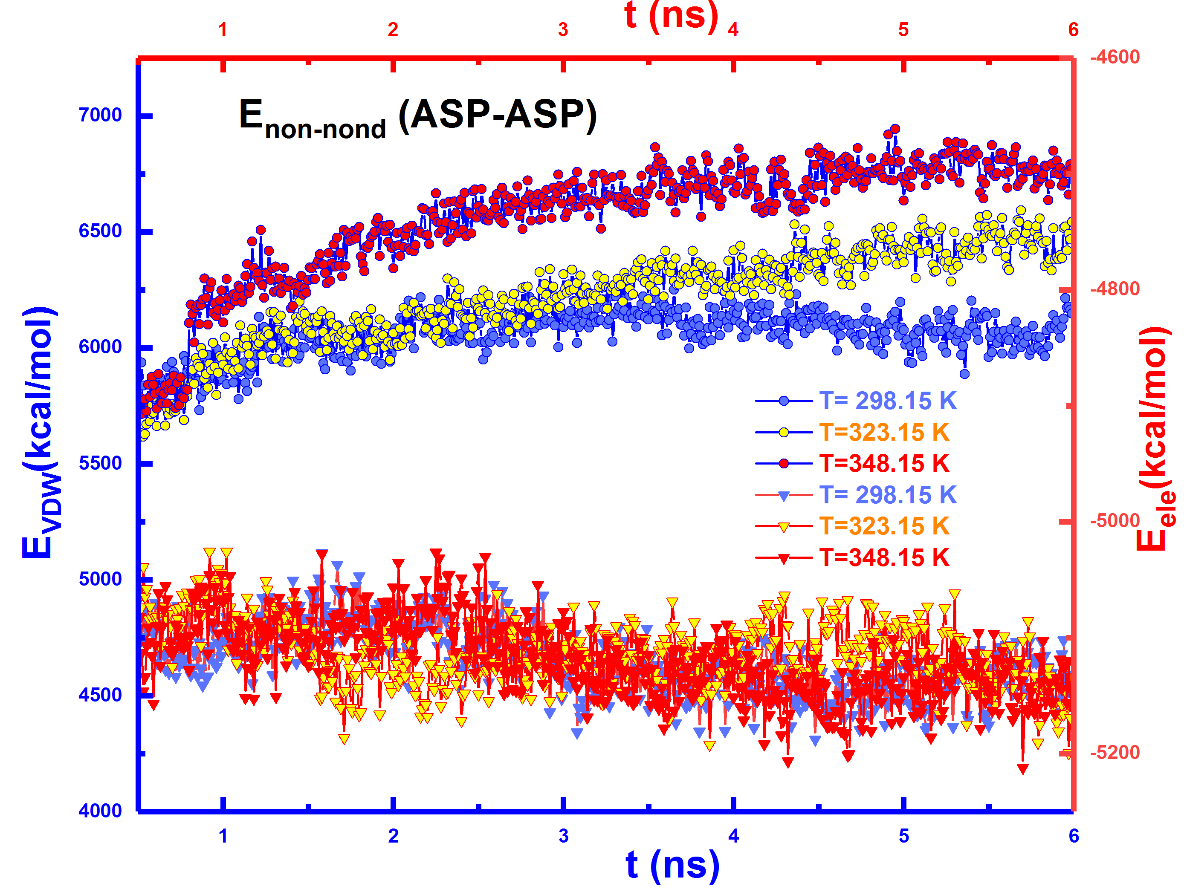

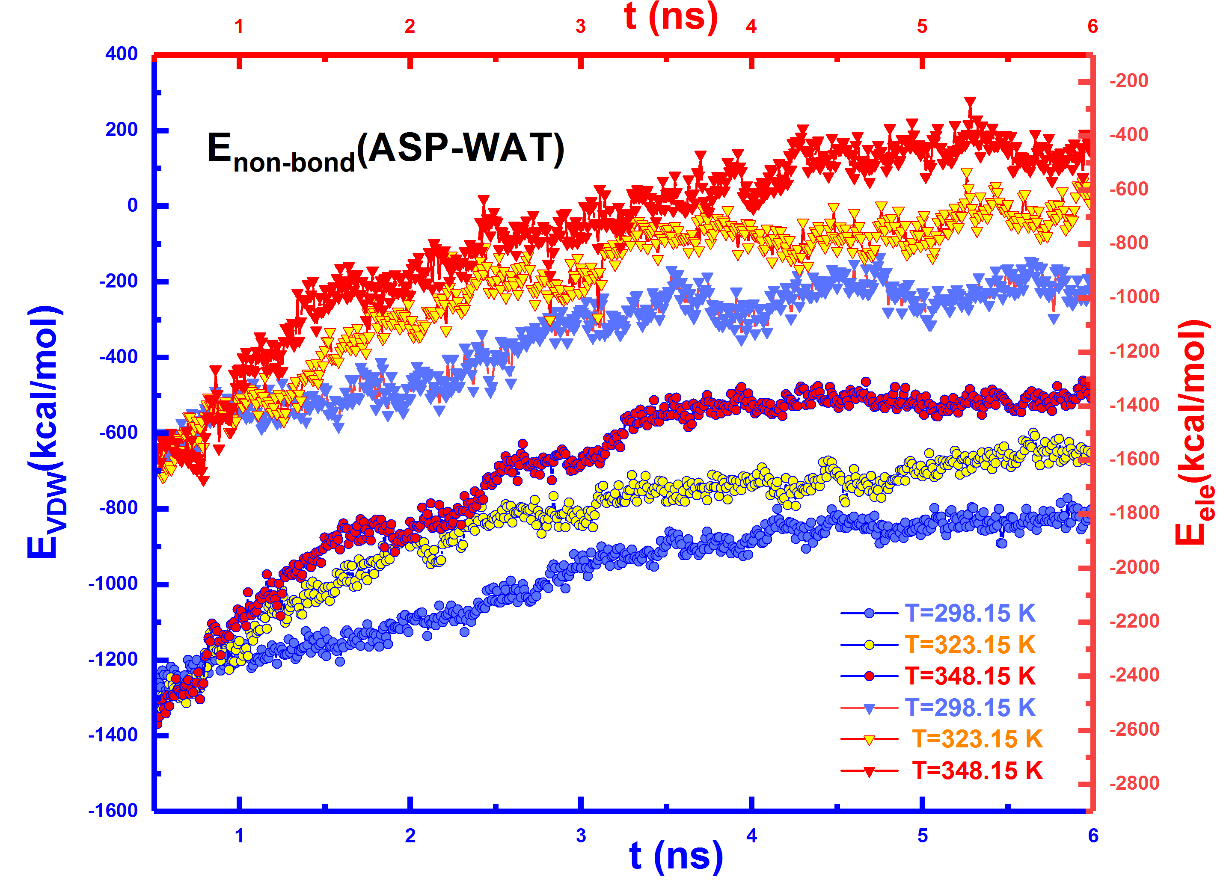


**(a)**

**(b)**


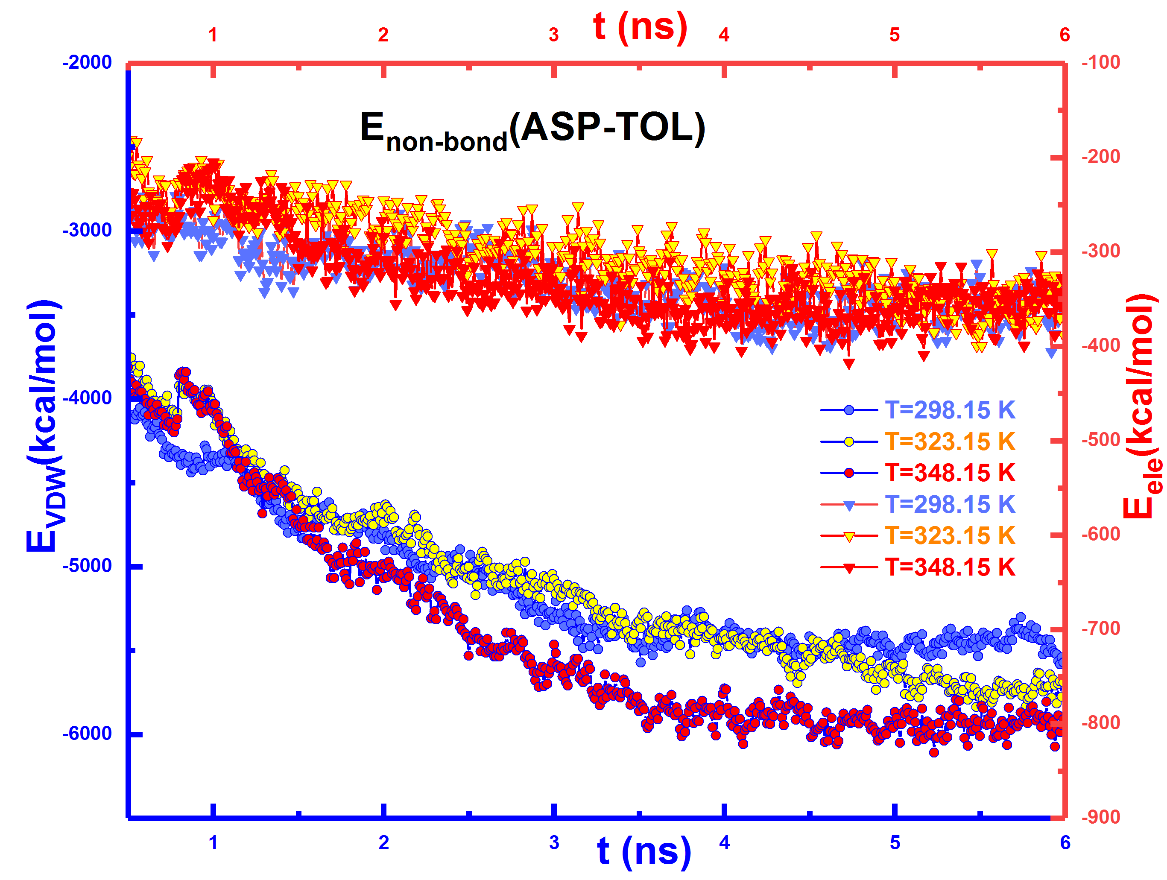

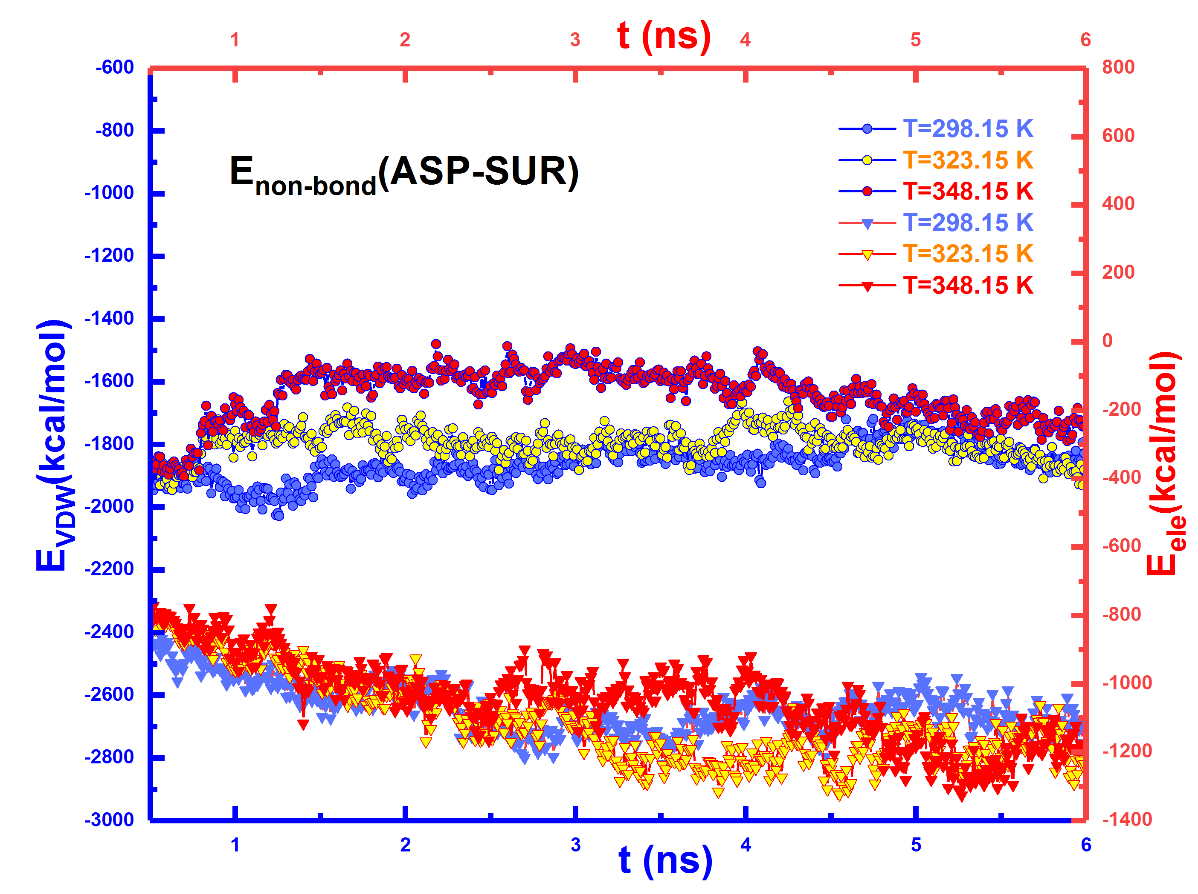


**(c)**

**(d)**

**Figure S5.** The plot of E_VDW_ and E_ele_ associated with some pair contents of the systems at different temperature (a,b,c,d).

**LUMO**

**HOMO**

**
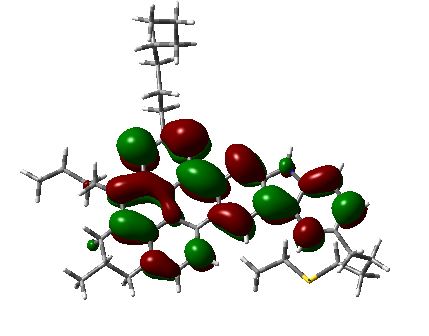

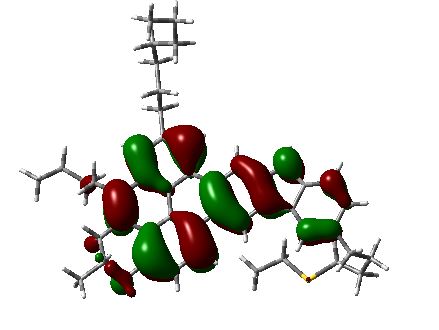
**

**a)**

**
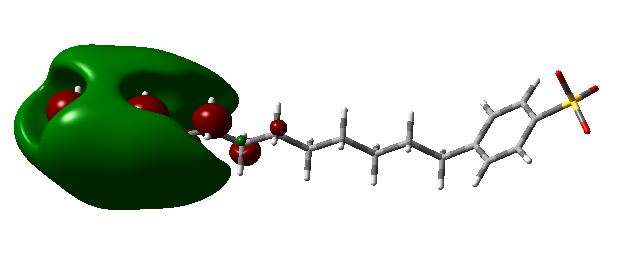

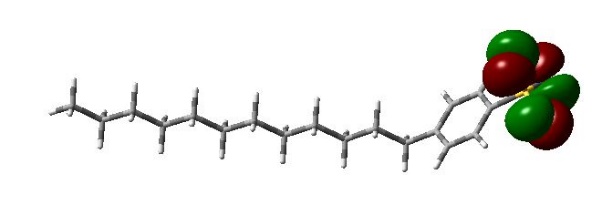
**

**b)**

**
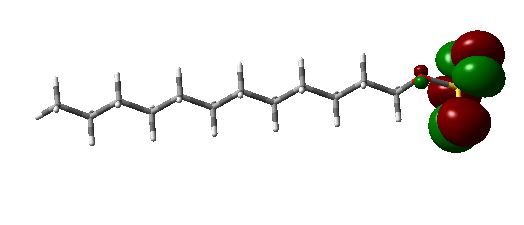

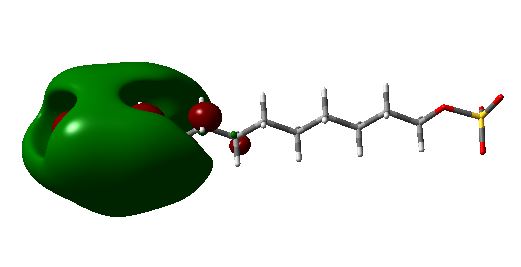
**

**c)**

**
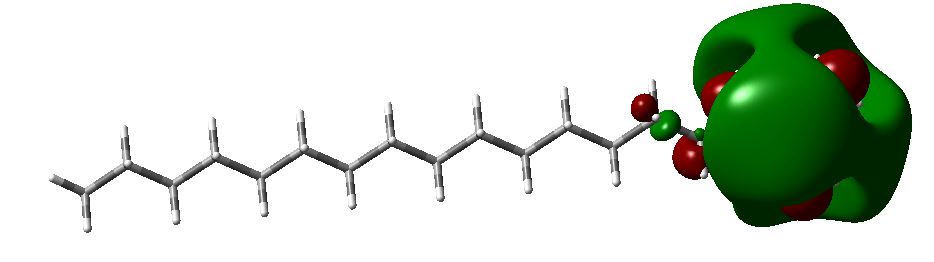

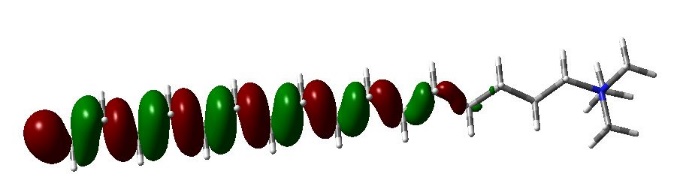
**

**d)**

**
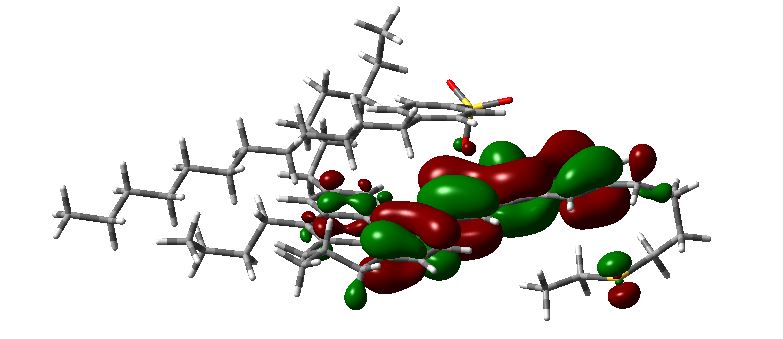

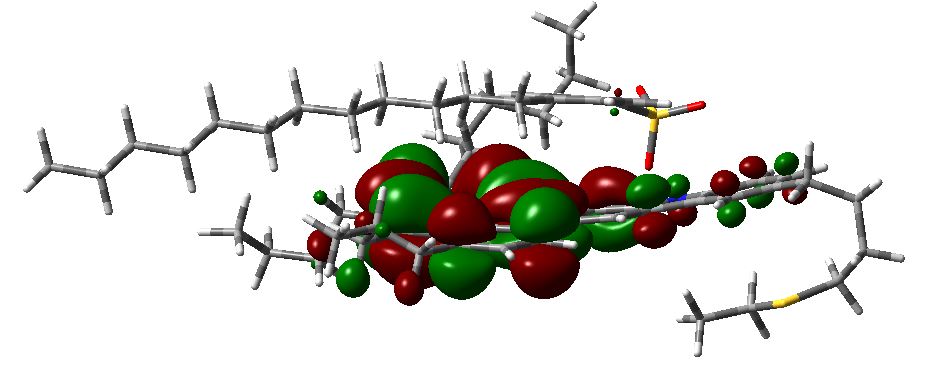
**

**e)**

**
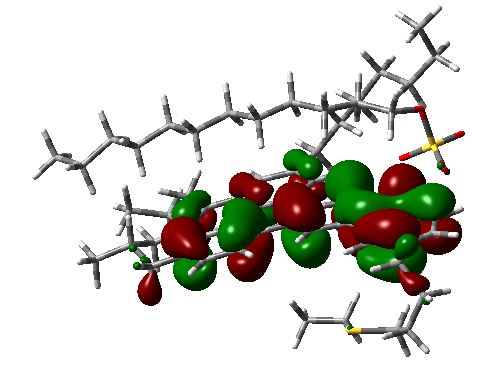

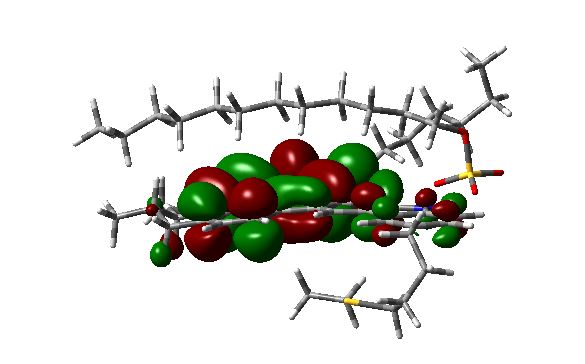
**

**f)**


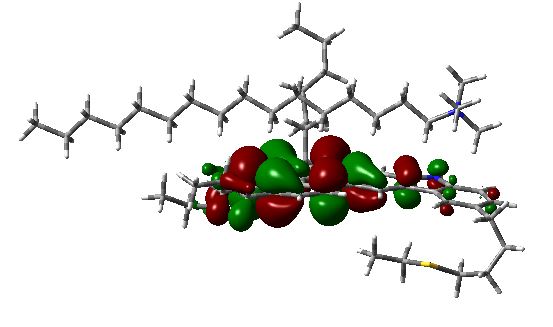

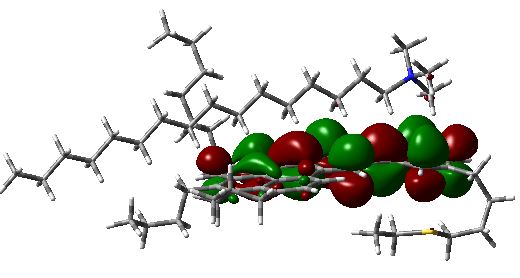


**g)**

**Figure S6**. The HOMO and LUMO for ASP (a), SDBS (b), SDS (c), CTAB (d), SDBS-ASP (e), and CTAB-ASP.

The HOMO orbitals of the anionic surfactants and CTAB are distributed in their head groups and tail, respectively. The distribution of HOMO and LUMO orbitals belonging to ASP and complexes are almost observable in benzene rings and ASP molecule, respectively.

| **Surfactant kind effect** | | | | | | |
| --- | --- | --- | --- | --- | --- | --- |
| **Surfactant name** | **Surfactant number** | **ASP number** | **Toluene number** | **Water number** | **Size box / (Å^3^)** | **T (K)** |
| **--------------** | **--------------** | **150** | **4500** | **4500** | **103.6×103.6×103.6** | **298.15** |
| **CTAB** | **200** | **150** | **4500** | **4500** | **107.4×107.4×107.4** | **298.15** |
| **SDS** | **200** | **150** | **4500** | **4500** | **106.0×106.0×106.0** | **298.15** |
| **SDBS** | **200** | **150** | **4500** | **4500** | **106.6×106.6×106.6** | **298.15** |
| **Concentration effect** | | | | | | |
| **Surfactant name** | **Surfactant number** | **ASP number** | **Toluene number** | **Water number** | **Size box / (Å^3^)** | **T (K)** |
| **SDBS** | **100** | **150** | **4500** | **4500** | **105.1×105.1×105.1** | **298.15** |
| **SDBS** | **200** | **150** | **4500** | **4500** | **106.6×106.6×106.6** | **298.15** |
| **SDBS** | **400** | **150** | **4500** | **4500** | **109.6×109.6×109.6** | **298.15** |
| **Temperature effect** | | | | | | |
| **Surfactant name** | **Surfactant number** | **ASP number** | **Toluene number** | **Water number** | **Size box / (Å^3^)** | **T (K)** |
| **SDBS** | **200** | **150** | **4500** | **4500** | **106.6×106.6×106.6** | **298.15** |
| **SDBS** | **200** | **150** | **4500** | **4500** | **108.0×108.0×108.0** | **323.15** |
| **SDBS** | **200** | **150** | **4500** | **4500** | **108.9×108.0×108.0** | **348.15** |

**Table S1**

Table S2. Characterization of crude oil

| **Properties** | **Value** |
| --- | --- |
| Viscosity (at 298K) | 37.7 (±0.2) cP |
| Saturate | 61.1% |
| Aromatic | 27.7% |
| Resin | 7.9% |
| Asphaltene | 3.3% |
| API | 17.4 |

Table S3. The interaction energy between water and other components.

| **compounds** | $\boldsymbol{-E (kJ.}\boldsymbol{mol}^{\boldsymbol{-1}}\boldsymbol{)}$ |
| --- | --- |
|  |  |
| SDBS-Water | 90 |
| SDS-Water | 73 |
| CTAB-water | 59 |
| ASP-Water | 37 |

Table S4. The hydrogen bonding between water and other components.

| compounds | Atoms | $\rho$ | $\nabla^{2}\rho$ | $E_{HB}(kj {mol}^{-1})$ | $r(nm)$ |
| --- | --- | --- | --- | --- | --- |
|  |  |  |  |  |  |
| SDBS-Water | $S-O\ldots H$ | 0.019 | 0.019 | 19.9 | 0.20 |
|  | $S-O\ldots H$ | 0.022 | 0.022 | 24.7 | 0.20 |
|  |  |  |  |  |  |
| SDS-Water | $S-O\ldots H$ | 0.020 | 0.018 | 19.9 | 0.21 |
|  | $S-O\ldots H$ | 0.022 | 0.021 | 22.7 | 0.20 |
|  |  |  |  |  |  |

Reference

1. Teklebrhan, R. B., Ge, L., Bhattacharjee, S., Xu, Z. & Sjöblom, J. Initial partition and aggregation of uncharged polyaromatic molecules at the oil--water interface: a molecular dynamics simulation study. *J. Phys. Chem. B* **118**, 1040–1051 (2014).

2. Rogel, E. Simulation of interactions in asphaltene aggregates.  *Energy Fuels* **14**, 566–574 (2000).
